# Association between antimicrobials administered and antimicrobial resistance profiles in food-producing animals in West Africa: a systematic review and meta-analysis

**DOI:** 10.1186/s12917-026-05971-1

**Published:** 2026-10-04

**Authors:** Djifahamaï Soma, Namwin Siourimè Somda, Fatimata B.J. Diarra, Djibril Diallo, Zakaria Garba, Souleymane Sore, Evariste Bako, Marguerite E.M. Nikiema, Nicolas Barro, Isidore J.O. Bonkoungou

**Affiliations:** 1 Université Joseph KI ZERBO, Ouagadougou, Burkina Faso; 2 Institut de Recherche en Sciences Appliquées et Technologies, Bobo-Dioulasso, Burkina Faso; 3 Clinical Research Unit of Nanoro, Ouagadougou, Burkina Faso; 4 Ministère de la Santé, Ouagadougou, Burkina Faso; 5 Centre Universitaire de Tenkodogo, Tenkodogo, Burkina Faso; 6 Institut de l’Environnement et de Recherches Agricoles, Ouagadougou, Burkina Faso; 7 Institut National de Santé Publique, Ouagadougou, Burkina Faso

**Keywords:** Antimicrobial resistance, antimicrobial use, Association, Food-producing animals, Livestock, West Africa

## Abstract

**Background:**

Antimicrobial resistance in farm animals is a growing threat to public health and food safety, particularly in West Africa, where antimicrobial use is often unregulated. The objective of this systematic review and meta-analysis is to evaluate the association between the antimicrobials administered in livestock farming systems (including both terrestrial livestock and aquatic species) and the antimicrobial resistance profiles reported in laboratory research studies in West Africa, assessing the alignment between field usage and laboratory surveillance data.

**Methods:**

This study was conducted as a systematic review and meta-analysis following the PRISMA 2020 protocol. This review involves an exhaustive literature search, the selection of studies based on predefined criteria, data extraction, and a qualitative and quantitative synthesis of the data. Studies published between 2015 and 2025 on antimicrobials use and antimicrobial resistance in food-producing animals, were included in this systematic review and meta-analysis. Articles were downloaded from PubMed, Scopus, African Journals Online (AJOL), and Google Scholar. Pooled antimicrobial resistance prevalence was estimated using random-effects metanalysis. Between-study heterogeneity was assessed using I² and Cochran’s Q test, whereas, publication bias was evaluated using funnel plots visualization and statistically tested using Egger’s regression test. The association between antimicrobial use and antimicrobial resistance was assessed using Spearman’s rank correlation. All analyses were performed using R (v4.5.2), and results were considered significant at *p* < 0.05.

**Results:**

This systematic review and meta-analysis included 102 eligible studies on antimicrobial resistance in food-producing animals. The single pooled antimicrobial resistance prevalence was estimated at 40% (95% CI: 35–45%) using a random-effects model, with substantial heterogeneity among studies (I² = 99.4%). The subgroup antimicrobial resistance prevalence was considerably substantial for sulphonamides (73%), tetracyclines (69%), penicillin (60%), and macrolides (55%), whereas but low for carbapenems (10%) and other antimicrobial classes. Antimicrobial use patterns varied according to animal species, with therapeutic use predominating in cattle and pigs, whereas prophylactic use was more frequently reported in poultry and small ruminants. In fish, therapeutic and prophylactic uses were reported at comparable levels. An ecological association was observed between reported antimicrobial use patterns and antimicrobial resistance profiles (Spearman’s ρ = 0.41; *p* = 0.013).

**Conclusions:**

This meta-analysis identifies an association between reported antimicrobial usage and antimicrobial resistance profiles in West African livestock. While this confirms a link between field practices and laboratory findings, data heterogeneity necessitates cautious interpretation. Our findings highlight the urgent need for standardized, coupled monitoring of antimicrobial consumption and resistance to inform robust regional control strategies.

**Supplementary Information:**

The online version contains supplementary material available at https://doi.org/10.1186/s12917-026-05971-1.

## Background

Antimicrobial resistance (AMR) is a major public health issue with significant impacts on human and animal health, as well as food safety in West Africa [1]. When bacteria develop resistance to an antimicrobial, the drug’s effectiveness is considerably reduced [2]. Bacteria acquire antimicrobial resistance through several mechanisms, including spontaneous genetic mutations and horizontal gene transfer mediated by mobile genetic elements such as plasmids, transposons, and integrons. These mechanisms enable bacteria to produce antimicrobial inactivating enzymes, modify antimicrobial targets, reduce drug uptake, or actively expel antimicrobial compounds, facilitating the persistence and dissemination of resistant strains within animal, human, and environmental reservoirs. This leads to an increase in disease prevalence and mortality, leading to rising healthcare costs [3].

AMR is a multifaceted phenomenon that has repercussions on human and animal health, as well as on the environment [4, 5]. Without effective antimicrobial stewardship and surveillance strategies, the global burden of AMR is expected to increase substantially in the coming decades, driven by rising antimicrobial consumption, population growth, and increasing demand for animal protein. This threat is particularly concerning in regions where veterinary services, diagnostic capacity, and regulatory frameworks remain limited. The use of antimicrobials in farm animals, particularly when administered incorrectly or inappropriately, is a major driver in the emergence and spread of antimicrobial resistant bacteria [6].

Livestock farming plays a central role in the global economy, with the African livestock sector contributing between 30% and 80% of the continent’s agricultural gross domestic product [7]. In several West African countries, cattle farming provides a livelihood for around one million producers and households, and contributes between $71 million and $115 million to the national economy [8]. This sector is of paramount importance, as it is vital for food security, income generation, and the preservation of cultural traditions. However, the use of antimicrobials in agriculture is not subject to rigorous monitoring. It is common for farmers to obtain veterinary medicines without a prescription and to determine the dosage or duration of treatment based on their own perception of effectiveness or the desired growth rate of the animal [9]. These habits facilitate the proliferation of resistant bacteria, which can have detrimental effects on human health, agricultural workers, and the environment [10].

Scientific knowledge regarding antimicrobial resistance in food-producing animals in West Africa is mainly based on two complementary types of studies: (i) antimicrobial use (AMU) surveys, which describe the types, frequency, indications, and target animal species of antimicrobial use [5, 11, 12], and (ii) antimicrobial resistance (AMR) studies, which investigate resistance profiles and the occurrence of resistant bacteria in food-producing animals [10, 13, 14]. While numerous studies have individually documented the prevalence of resistant bacteria in animals or provided descriptions of antimicrobial use on farms, there is a persistent lack of synthesized evidence that bridges these two domains. Most existing literature treats veterinary field practices and laboratory-confirmed resistance phenotypes as isolated phenomena. Consequently, there is an urgent need to consolidate this disparate body of evidence to evaluate the relationship between documented antimicrobial administration and the resistance patterns identified through laboratory screening, thereby establishing a clearer epidemiological link that can inform effective regional control strategies. The objective of this systematic review and meta-analysis is to evaluate the association between the antimicrobials administered in livestock farming systems (including both terrestrial livestock and aquatic species) and the antimicrobial resistance profiles reported in laboratory research studies in West Africa, assessing the alignment between field usage and laboratory surveillance data.

## Methods

### Study design and protocol

This study was registered in the Open Science Framework with registration DOI: https://doi.org/10.17605/OSF.IO/32EDT. This study was conducted as a systematic review following a predefined protocol. The review process involved an exhaustive literature search, the selection of studies based on predefined eligibility criteria, data extraction, a quantitative and qualitative synthesis of the results. The results of the review were presented in accordance with the PRISMA 2020 guidelines (Preferred Reporting Items for Systematic Reviews and Meta-Analyses) [15].

### Search strategy

To structure this systematic review and meta-analysis, the research question was defined according to the PEO (Population, Exposure, Outcome) framework, which is appropriate for observational epidemiological studies:

- Population (P): Food-producing animals, including terrestrial livestock and aquatic species, in West Africa.
- Exposure (E): Antimicrobial use practices, including the types of antimicrobials used, indications, frequency of administration, and management practices in farming systems.
- Outcome (O): Antimicrobial resistance profiles, including the prevalence of resistant bacteria and resistance patterns identified through laboratory-based investigations.

A comprehensive literature review was conducted of studies published between January 2015 and December 2025. This period was chosen in order to gather recent data following the launch, in 2015, of the WHO Global Action Plan on Antimicrobial Resistance, which has facilitated the establishment of a standardised surveillance and reporting system in the region using four electronic databases: PubMed, Scopus, African Journals Online (AJOL) and Google Scholar. Database-specific search fields were considered according to the search interfaces of each platform. Searches were conducted using the default search fields available in each database, with the corresponding keywords and Boolean operators. The complete search strategies, including the database-specific search fields, keywords, and Boolean operators, are provided in Supplementary Table 1. The titles, abstracts and full texts of the retrieved articles were reviewed to verify their compliance with the predefined eligibility criteria. Only studies reporting antimicrobials use and resistance in food-producing animals in West Africa and published in English or French were included.

### Eligibility criteria

#### Inclusion criteria

- Studies conducted in West African countries.
- Studies reporting antimicrobials use in livestock farming (as reported by farmers) or AMR results from bacteria isolated from livestock.
- Studies reporting both antimicrobials use and AMR results.
- Primary studies published in English or French between 2015 and 2025.

#### Exclusion criteria

- Conference abstracts, review articles, letters to the editor, case reports, research notes, or systematic reviews.
- Laboratory studies focusing solely on the transfer of resistance genes.
- Studies conducted during epidemics.
- Studies focusing exclusively on the genotypic identification of bacteria.
- Studies conducted outside of West Africa.
- Molecular studies unrelated to livestock farming.
- Studies lacking antibiogram data.
- Duplicate data reported in publications.
- Studies combining data from West Africa and other regions without disaggregation.
- Studies reporting AMR only as an overall summary for multiple species without species-specific and AMR-specific data.
- Studies having tested less than a minimum of 10 bacterial isolates.

#### Screening and calibration process

- Title and abstract screening phase: Records were screened independently by two reviewers against the predefined eligibility criteria. Specifically, studies were included if they: (1) were conducted in West African countries, (2) targeted food-producing animals, and (3) reported quantitative data on antimicrobial usage or antimicrobial resistance profiles. Studies were excluded if they were case reports, reviews, or lacked specific data on the West African context. Studies that clearly failed to meet the inclusion criteria were excluded.
- Full-text assessment phase: The full texts of the retained articles were evaluated independently by the same reviewers.
- Calibration exercise: Prior to the formal screening process, a calibration exercise was conducted using a random sample of 10% of retrieved records to ensure consistent interpretation and application of the eligibility criteria among reviewers. Discrepancies identified during this exercise were discussed and resolved before proceeding with the full screening.

### Assessment of study quality

To ensure the rigour of this review, two reviewers independently assessed the methodological quality of the selected publications using the Joanna Briggs Institute (JBI) critical appraisal tool adapted for prevalence studies [16]. The review focused on nine indicators, covering in particular the appropriateness of the sampling strategy, the accuracy of the description of the context and participants, and the validity of the measurement instruments and statistical methods used. Each criterion was scored on a binary scale, with one point awarded for a positive response and zero for a negative or Unclear response (supplementary Table 4). Disagreements between the reviewers, DS. and NSS., were resolved through consultation or, where necessary, through the intervention of FBJD. and IJOB. The total quality score ranged from 0 to 9 points. Studies were classified as high quality (7–9 points), moderate quality (5–6 points), or low quality (≤ 4 points). Studies classified as low quality (≤ 4 points) were excluded from the quantitative synthesis. Following this assessment, all included studies achieved scores ranging from 7 to 9 points, corresponding to high methodological quality.

### Data extraction

Data were extracted independently by DS, NSS and FBJD using a standardized data extraction form developed for this systematic review. The extracted variables were grouped into three main categories: (i) study characteristics, including first author, year of publication, country, study design, and study type (antimicrobial use (AMU), antimicrobial resistance (AMR), or both); (ii) animal and sampling characteristics, including animal species, production system, sample type, sample source, and sample size; and (iii) microbiological and antimicrobial-related characteristics.

For AMU studies, extracted information included antimicrobial classes and antimicrobial used, purpose of use (therapeutic, prophylactic, or growth promotion), source of AMU data (farmer surveys, farm records, or sales data), veterinary supervision status, and diseases targeted. For AMR studies, extracted variables included bacterial species identified, antimicrobial susceptibility testing methods (disk diffusion, MIC, or genotypic methods), antimicrobials tested, number of isolates tested, number of resistant isolates, and resistance percentages. When available, molecular data, including resistance genes and mobile genetic elements, were also collected. All extracted data were entered into Microsoft Excel for subsequent analysis.

### Data analysis

All statistical analyses were performed using R software (version 4.5.2) with the meta and dplyr packages. The AMR dataset was imported from a structured csv file and pre-processed prior to analysis. Study identifiers (Study.ID) were verified and, when missing, unique identifiers were automatically assigned to enable consistent aggregation and labelling.

For the meta-analysis of antimicrobial resistance, data were aggregated by study and by antimicrobials class, adding the number of resistant isolates (N_Resistant) and the total number of isolates tested (N_Tested) within each group. This approach allowed us to estimate pooled resistance proportions both globally and separately for each antimicrobial class. A meta-analysis of proportions was performed using the metaprop function of the meta package. Resistance proportions were analysed using a logit transformation (sm = “PLOGIT”) to stabilize variances. Studies were combined using the inverse variance weighting method. Random effects models were used throughout the study to account for expected heterogeneity between studies resulting from differences in study design, animal species, countries, and laboratory methods.

Forest plots were generated using the forest() function from the metafor package for the global data set and separately for each antimicrobial class, displaying individual study estimates and pooled resistance proportions with 95% confidence intervals. Plot dimensions, font sizes, and study labels were adjusted to improve readability. Funnel plots were produced using the funnel() function of the metafor package in R. for the overall analysis and for each antimicrobial class to visually assess potential publication bias. Heterogeneity between studies was quantified using I² and τ² statistics, and the statistical significance of heterogeneity was assessed using Cochran’s Q test.

To explore the relationship between antimicrobial use and antimicrobial resistance, a Spearman rank correlation analysis was performed between the frequency of antimicrobial use reports and the corresponding resistance frequencies reported in the included studies. Because antimicrobial use data were derived from reported frequencies rather than standardized quantitative consumption indicators, this analysis was considered an ecological assessment of association and not a measure of causal effect.

The frequency of antimicrobial occurrence was calculated by aggregating the number of records for each distinct antimicrobial agent within the study databases. Antimicrobials were classified into three categories: common agents present in both testing and usage data, agents specific to antimicrobial AMR panels, and agents specific to AMU records. Bar charts were used to illustrate the distribution of antimicrobials across different categories (common to AMU and AMR, specific to AMU, and specific to AMR), with a distinct colour code for each category. In addition, hexagonal binning plots were generated using the geom_hex() function of the ggplot2 package in R to visualize the relationship between antimicrobial use and resistance. For the hexagonal binning plots, the parameter bins = 50 was applied within the geom_hex() function. This partitioned the plotting area into 50 equal hexagonal cells along both axes, allowing for an optimal balance between data granularity and the identification of density patterns.

To map and compare resistance hotspots across the different animal species for the heatmap, data analysis was performed by filtering the raw data to retain only pathogen-antimicrobial combinations with a resistance percentage greater than 20%, thereby focusing on significant resistance levels of epidemiological interest. The data were then aggregated by animal species, pathogen, and antimicrobial class tested by calculating the arithmetic mean of their resistance percentages.

Descriptive statistics, including means and standard deviations, were calculated to summarize the percentage of antimicrobial use across species and administration purposes. A Kruskal-Wallis test was applied to evaluate the significance of differences between therapeutic, prophylactic, and growth promotion uses. All analyses were two-tailed, and results were considered statistically significant at *p* < 0.05.

The geographical distribution of the included studies across West African countries was mapped using QGIS version 3.28.14 (QGIS Development Team).

## Result

### Selection of studies

**Fig. 1:**
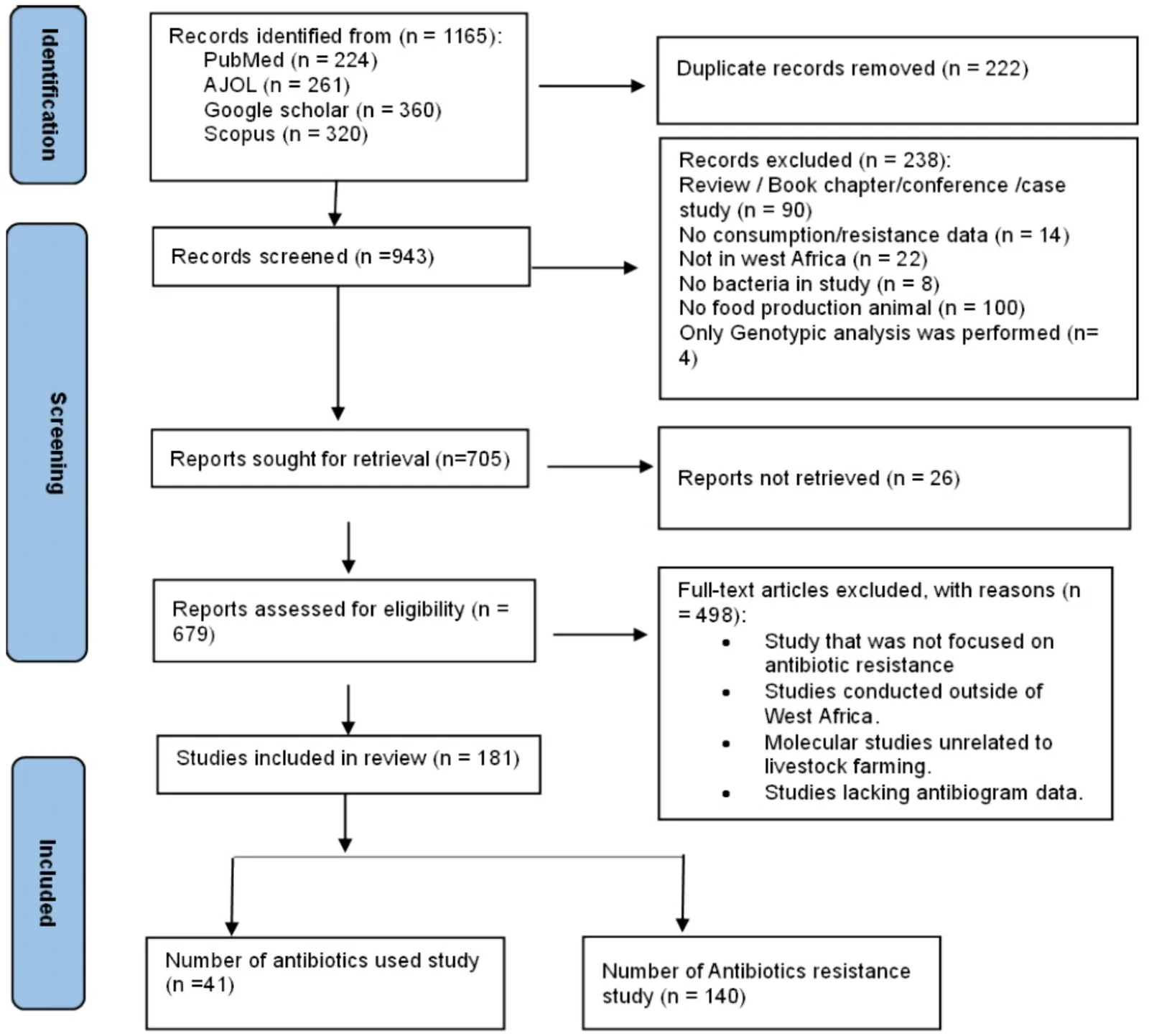
PRISMA flowchart of study selection

The initial search identified 1,165 records from four databases. After removing duplicates (*n* = 222), a total of 943 records were screened, leading to the exclusion of 238 records during the initial screening phase based on our exclusion criteria. Exhaustive details regarding these reasons for exclusion, as well as subsequent full-text exclusions (*n* = 498) are presented in Fig. 1. Following this assessment, 181 studies were included in the systematic review, comprising 140 studies on antimicrobial resistance and 41 on antimicrobial use. Of these, 20 studies reported both AMR and AMU data. For the quantitative synthesis, only studies providing sufficient data for meta-analysis were retained. Consequently, 102 AMR studies and 21 AMU studies met the eligibility criteria and were included in the meta-analysis. The remaining studies were retained for the qualitative synthesis but excluded from the quantitative synthesis because they did not meet the predefined eligibility criteria, including an insufficient number of isolates tested (fewer than 10 isolates per bacterial species). Accordingly, the JBI quality assessment presented in Supplementary Table 4 includes only the studies retained for the quantitative meta-analysis.

### Characteristics of included studies

**Fig. 2:**
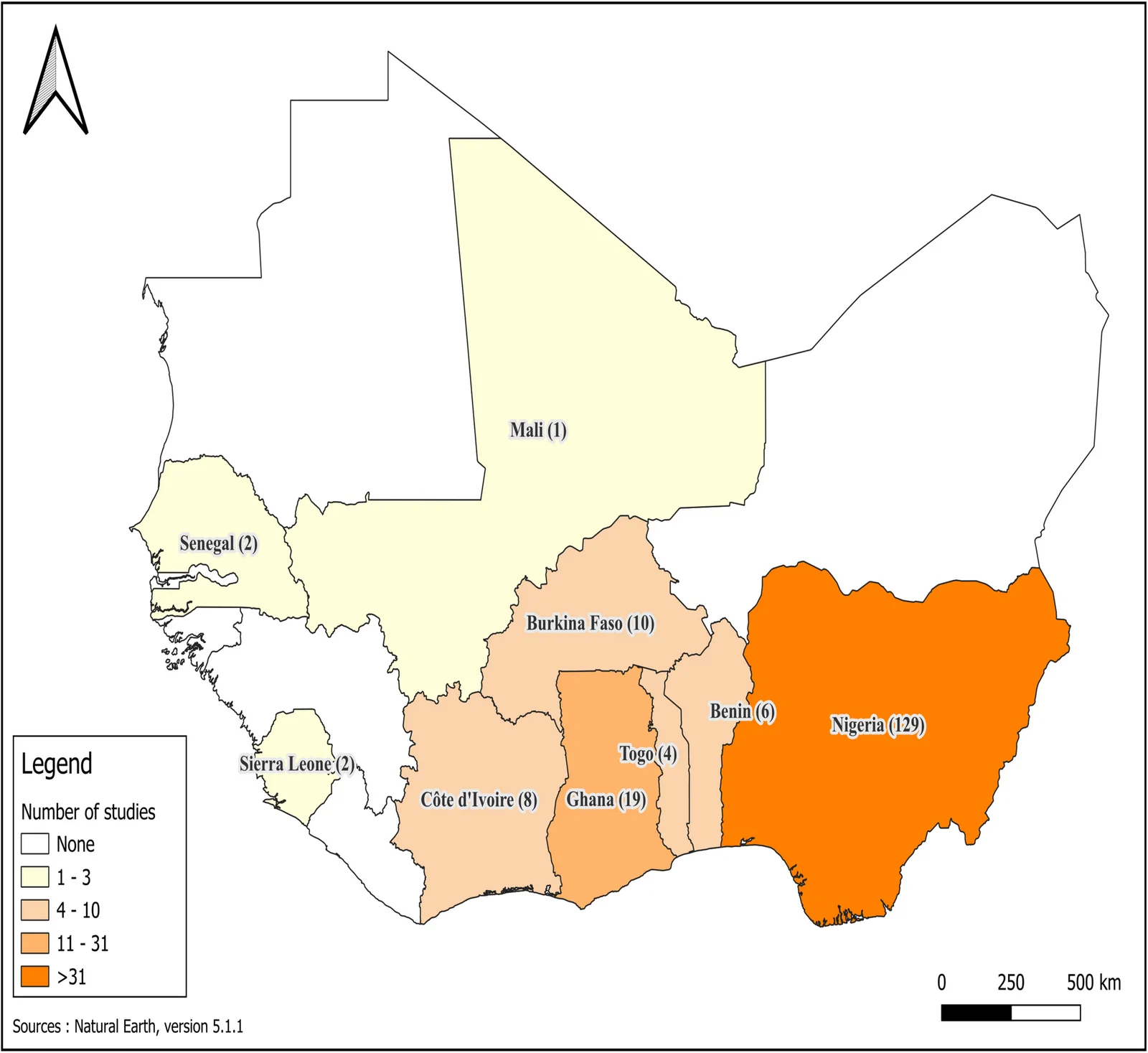
Geographical distribution of reviewed studies. Countries have been coloured based on the number of reported studies included in this review

The final selection comprised 181 studies published between 2015 and 2025. All included investigations utilized a cross-sectional study design. As shown in Fig. 1, the study selection process resulted in the inclusion of 181 studies and revealed a marked geographical disparity within West Africa, with only 9 of the 15 countries in the sub-region (60%) meeting the inclusion criteria. Nigeria contributed the largest number of studies included in the review with 129 studies (71.27%), followed by Ghana with 19 studies (10.49%), Burkina Faso with 10 studies (5.52%) and Côte d’Ivoire with 8 studies (4.41%). The other countries, notably Benin (*n* = 6; 3.31%), Togo (*n* = 4; 2.20%), Senegal (*n* = 2; 1.10%), Sierra Leone (*n* = 2; 1.10%) and Mali (*n* = 1; 0.55%), made a more limited contribution (Fig. 2). With regard to animal species, studies on antimicrobial use mainly target poultry (*n* = 15; 36.58%), cattle (*n* = 9; 21.95%) and sheep (*n* = 8; 19.51%). Furthermore, data on antimicrobial resistance show a marked predominance of research carried out on poultry (*n* = 70; 49.64%), cattle (*n* = 30; 21.27%) and pigs (*n* = 20; 14.18%). In terms of bacterial isolation analysis, *E. coli* was identified as the most commonly documented agent (*n* = 65; 35.71%). It is followed by *Salmonella* spp. (*n* = 29; 15.93%), *Klebsiella* spp. (*n* = 8; 4.39%) and *Klebsiella pneumoniae* (*n* = 6; 3.29%). Other pathogens such as *Staphylococcus aureus* and MR*SA* each account for (*n* = 5; 27.47%) of isolates out of a total of 182 identified across these specific studies.

### Comparative analysis of antimicrobial use and resistance

**Fig. 3:**
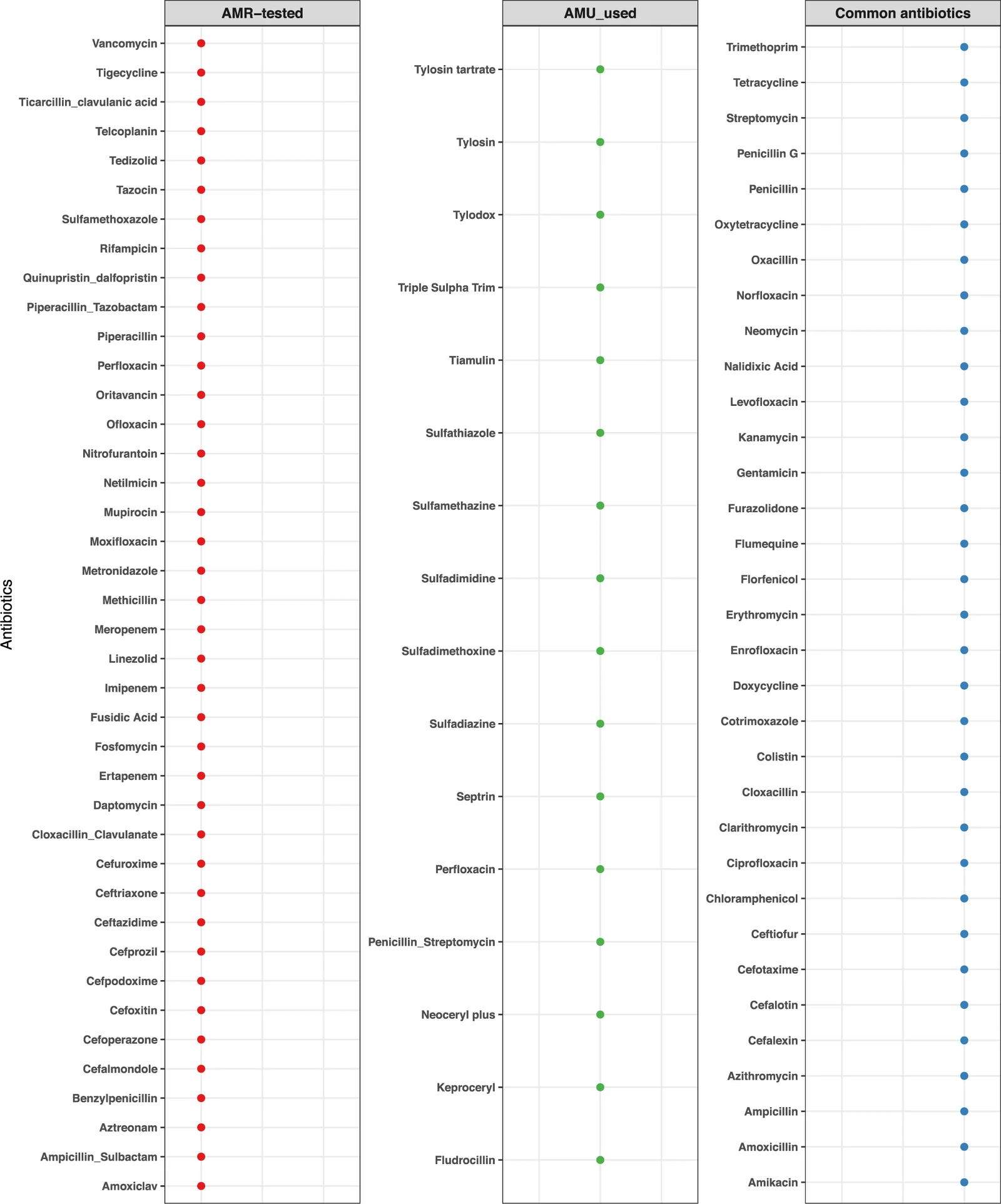
Distribution of individual antimicrobials identified in the included studies. Antimicrobials are presented individually to highlight antimicrobials -level overlap between antimicrobial use (AMU) and antimicrobial resistance (AMR) datasets. Antimicrobials were classified into three categories: (i) AMR-tested antimicrobials (**A**), reported only in AMR studies; (ii) AMU-reported antimicrobials (**B**), reported only in AMU studies; and (iii) common antimicrobials (**C**), reported in both AMU and AMR studies. This representation was chosen to facilitate direct comparison of individual antimicrobials rather than pharmacological classes

A total of 47 individual antimicrobials were identified across the included studies. These antimicrobials were classified according to whether their use was reported in antimicrobial use (AMU) studies, their resistance was assessed in antimicrobial resistance (AMR) studies, or both. This classification was used to assess the concordance between antimicrobials reported as being used in livestock production and those included in laboratory-based resistance surveillance. Three categories were identified. The AMR-tested only group (Fig. 3A) comprises antimicrobials included in AMR surveillance despite not being reported in AMU studies, reflecting precautionary monitoring of critically important antimicrobials such as carbapenems and vancomycin. The AMU-reported only group (Fig. 3B) includes antimicrobials reported in livestock production but not evaluated in AMR studies, highlighting potential gaps in resistance surveillance. This group includes tylosin, tiamulin, and several sulphonamides. The Common group (Fig. 3C) includes antimicrobials that were both reported in AMU studies and tested in AMR surveillance, indicating alignment between field use and laboratory monitoring. These include commonly used first-line agents such as amoxicillin, tetracyclines, and gentamicin.

### Purposes of antimicrobial use in food-producing animals

**Fig. 4:**
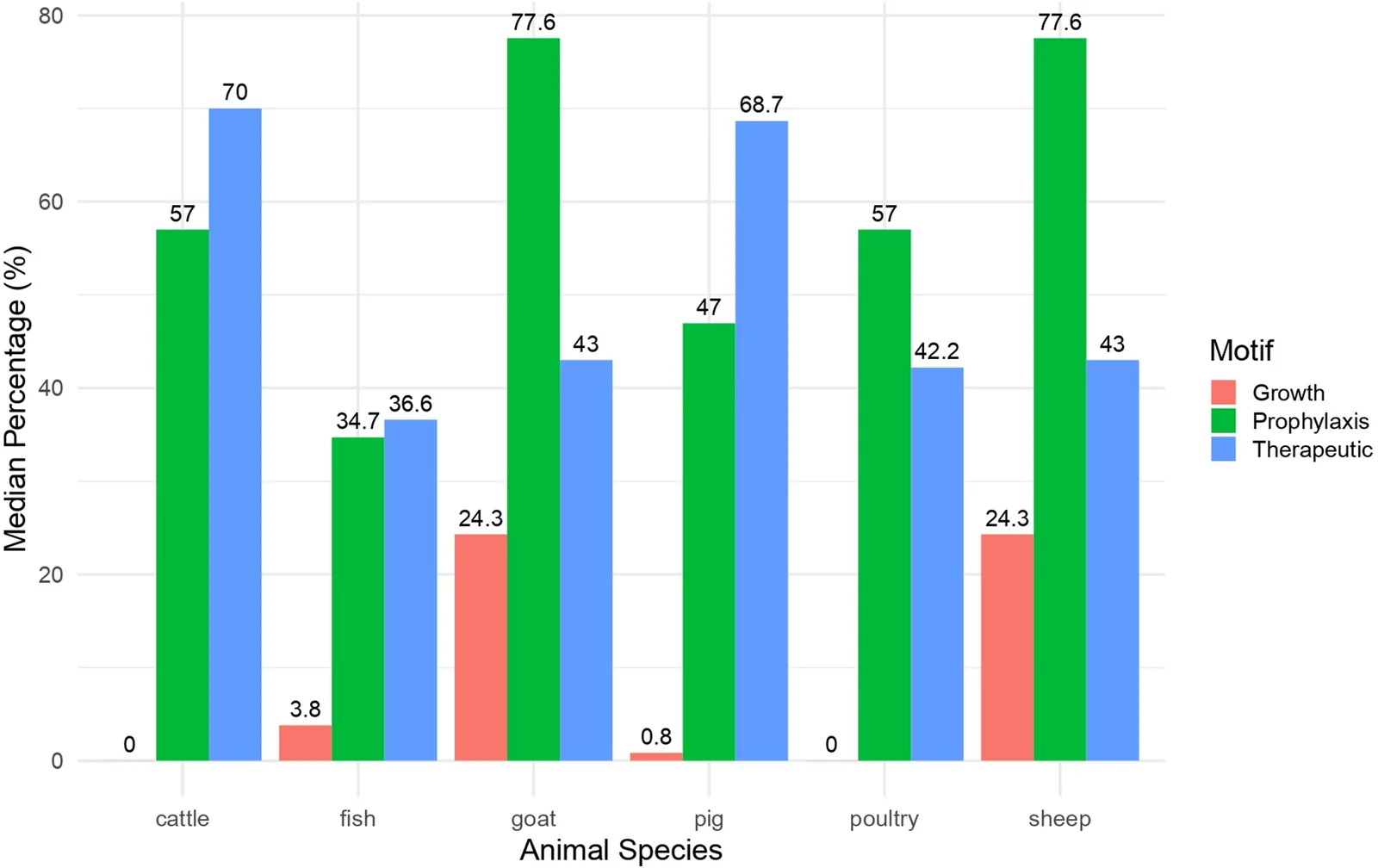
Median percentage of antimicrobial use by animal species and purpose. Bars represent the median percentage of antimicrobial consumption based on the reported purpose (Growth in red, Prophylaxis in green, Therapeutic in blue) for each animal species (cattle, fish, goat, pig, poultry, sheep). Numeric values above the bars indicate the medians calculated from data aggregated by study

Antimicrobial use patterns varied across animal species and intended purposes (Fig. 4). Overall, prophylactic use was the most frequently reported purpose in goats, sheep, poultry, and fish, whereas therapeutic use predominated in cattle and pigs. The use of antimicrobials as growth promoters was generally infrequently reported across all animal species.

Among cattle, therapeutic use accounted for 70% of reported antimicrobial use, followed by prophylactic use (57%), while no growth-promoting use was reported. Goats and sheep displayed similar patterns, with prophylactic use representing the highest proportion (77.60%), followed by therapeutic use (43%) and growth promotion (24.3%). In pigs, therapeutic use (68.70%) exceeded prophylactic use (47%), whereas growth promotion represented only 0.8% of reported use. Poultry showed predominantly prophylactic use (57%), followed by therapeutic use (42.20%), with no reported growth-promoting use. In fish, prophylactic and therapeutic uses were reported at comparable levels (34.7% and 36.60%, respectively), while growth promotion represented 3.80% of reported use.

Despite these descriptive differences, post-hoc pairwise comparisons using Dunn’s test with Bonferroni correction did not identify statistically significant differences between antimicrobial use purposes (growth promotion, prophylaxis, and therapeutic use) within animal species (adjusted *p* > 0.05) (Fig. 4; Supplementary Table 2).

### Distribution of bacterial species reported across the included studies

**Fig. 5:**
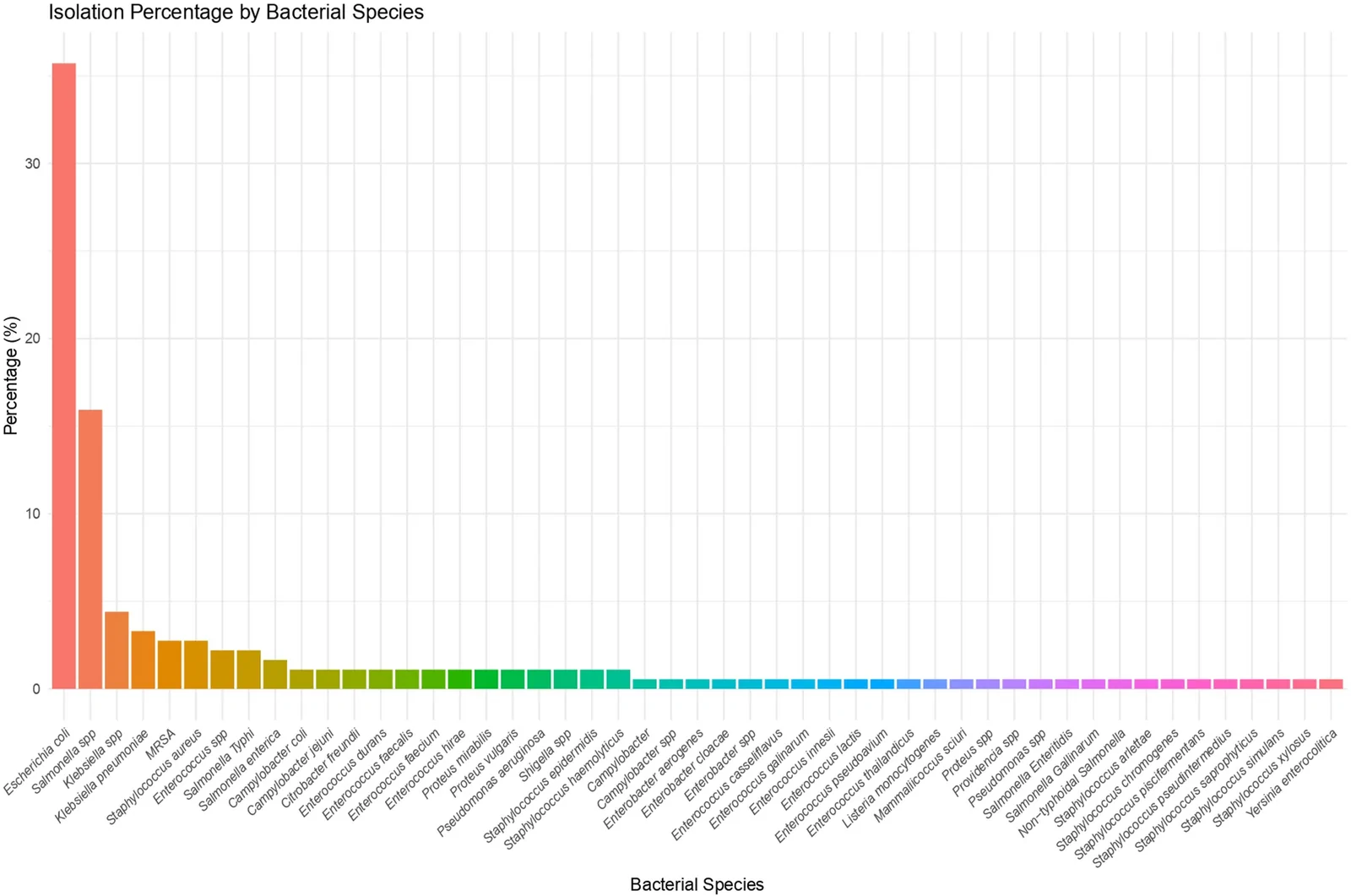
Distribution of bacterial species reported across the included studies. MRSA: Methicillin-Resistant *Staphylococcus aureus*

A total of more than 40 bacterial species or species groups were reported across the included studies. Among the bacterial species reported, *Escherichia coli* was the most frequently identified, representing 35.71% (65/182) of all bacterial records extracted from the included studies., followed by *Salmonella* spp. (29/182; 15.93%) and several identified species and groups (*S. enterica*,* S.* Enteritidis, *S.* Gallinarum, *S.* Typhi, and Non-typhoidal *Salmonella*) (Fig. 5; Supplementary Table 3).

Other Gram-negative bacteria included opportunistic Enterobacterales such as *Klebsiella* spp (4.40%), *K. pneumoniae* (3.3%), *Proteus* spp (2.75%), *Citrobacter freundii* (1.10%), as well as *Pseudomonas* and *Providencia* species, all occurring at low frequencies (< 1.50%).

Gram-positive bacteria were also identified. The *Enterococcus* genus comprised several species, including *E. faecalis*, *E. faecium*, *E. durans*, and *E. hirae*. Specifically, unidentified *Enterococcus* spp. represented 2.20% of the reported bacterial records, while other minor species accounted for between 0.55% and 1.10%. Coagulase-positive and coagulase-negative staphylococci were also represented, notably *Staphylococcus aureus* and methicillin-resistant *Staphylococcus aureus* (MRSA) (2.75% each), as well as various other species of the genus *Staphylococcus* at low frequency (with each species accounting for ≤ 1.10% of the total isolates) (Fig. 5; Supplementary Table 3). Finally, bacteria of public health interest, such as *Listeria monocytogenes*,* Campylobacter* spp, *Yersinia enterocolitica*, and *Shigella* spp, were identified but in very low proportions (0.55–1.10%) **(**Fig. 5; Supplementary Table 3).

### Antimicrobial resistance by animal species

**Fig. 6:**
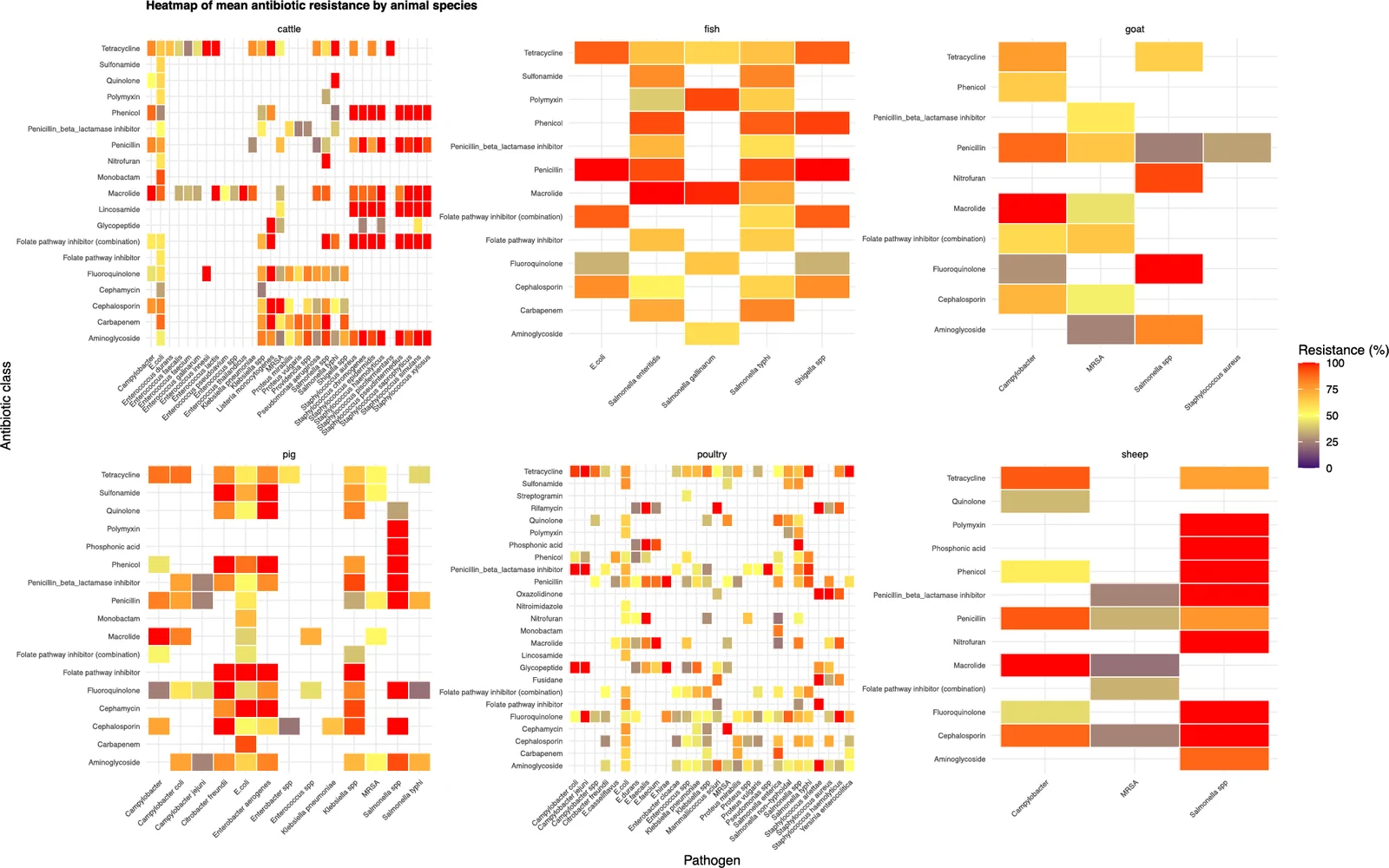
Heatmap showing mean antimicrobial resistance (%) by tested antimicrobial class and pathogen across different animal species. Each facet represents a specific animal species. Colours indicate the level of resistance: dark purple = low resistance, yellow = moderate resistance, bright red = high resistance

The antimicrobial resistance profiles of bacterial isolated from the different animal species studied are summarized in the heatmap (Fig. 6). In cattle, the predominant enterobacteria, notably *E. coli*, *Salmonella* spp. and *Klebsiella pneumoniae*, as well as *Enterococcus* spp., show a high resistance levels to tetracyclines, penicillin, macrolides and cephalosporins, ranging from 50% to 100%. Conversely, aminoglycoside resistance levels are lower (< 25%) and more heterogeneous across bacterial species, while carbapenems retain largely preserved activity (< 25%). In pigs, *E. coli*,* Salmonella spp.*,* Enterobacter* spp., and *Klebsiella* spp. are characterized by marked resistance to tetracyclines (75–100%), sulphonamides (75–100%), macrolides (75–100%), and fluoroquinolones (75–100%). Beta-lactams, including penicillin (75–100%) and cephalosporins (50–75%), show reduced efficacy across several pathogens due to the presence of resistance. In comparison, resistance to aminoglycosides and carbapenems remains limited (< 25%). In poultry, a wide variety of pathogens are observed, including *E. coli*,* Salmonella* spp., *Campylobacter* spp., *Staphylococcus* spp., *Enterococcus* spp., and *Proteus* spp. The highest levels of resistance are to tetracyclines (75–100%), penicillin (75–100%), macrolides (75–100%), fluoroquinolones (75–100%), and folate pathway inhibitors (75–100%). Cephalosporins show intermediate (50%) to high (75%) resistance profiles, while aminoglycosides and carbapenems remain associated with low levels of resistance (< 25%). Regarding the sheep farm, data mainly concern *Salmonella* spp., *Campylobacter* spp., and *Staphylococcus aureus*. These pathogens show significant resistance to tetracyclines (75–100%), penicillin (75–100%), macrolides (75–100%), and fluoroquinolones (75–100%), while cephalosporins show intermediate levels of resistance (50%). Aminoglycosides appear to be more effective (< 25%). In goats, *Salmonella* spp., *Campylobacter* spp., and *Staphylococcus aureus* exhibited high levels of resistance, particularly to penicillin (75–100%) and macrolides (75–100%). fluoroquinolone resistance varies among certain species (50–75%), while tetracyclines and cephalosporins show intermediate resistance levels (50%); aminoglycosides remain more effective (< 25%). Regarding the fish farm, the pathogens identified, including *E. coli*,* Salmonella* Enteritidis, *Salmonella* Typhimurium, and *Shigella* spp., show high resistance to penicillin (80–100%), tetracyclines (75–100%), macrolides (80–100%), and sulphonamides (75–90%). Cephalosporin and fluoroquinolone resistance levels vary from intermediate (50–75%) to high (75%), while aminoglycosides and carbapenems remain associated with generally preserved activity (< 25%).

## Meta-analysis

### Pooled prevalence of AMR in food producing animal

**Fig. 7:**
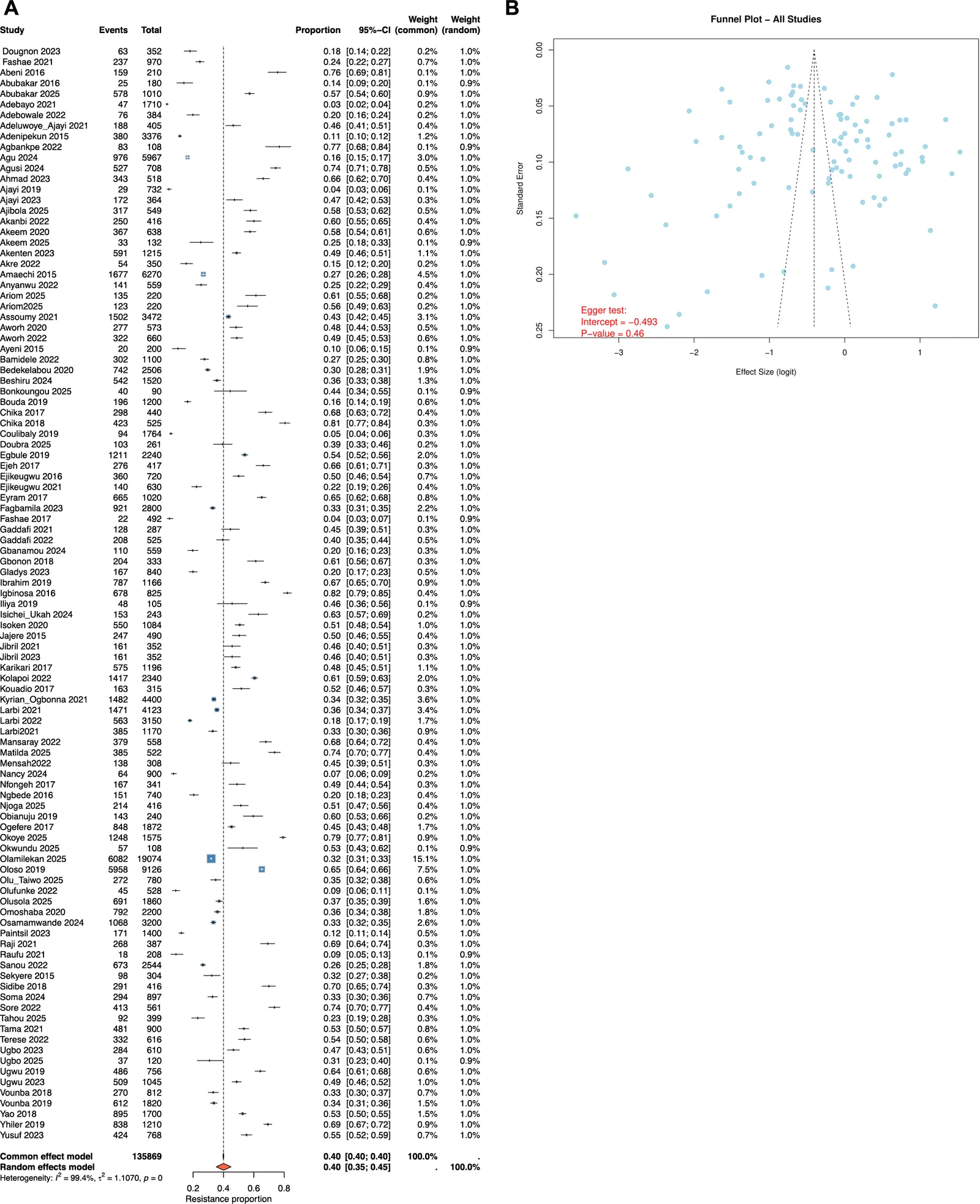
Global meta-analysis of antimicrobial resistance in food-producing animals. Panel **A** (Forest plot): Each square represents the proportion of resistant isolates per study, with size proportional to study weight. Horizontal lines show 95% confidence intervals. The red diamond represents the overall pooled estimate from the random-effects model. Panel **B** (Funnel plot): Standard error versus logit-transformed effect size for each study. Blue points represent individual studies. Symmetry indicates low publication bias. Egger’s regression test results (intercept and p-value) are shown in red at the bottom-left of the plot

The overall pooled prevalence of antimicrobial resistance was estimated at 40% (95% CI: 35–45%) using a random-effects model. The fixed-effects model yielded a comparable pooled estimate (40%; 95% CI: 40–40%). Very high heterogeneity was observed among the included studies (I² = 99.40%; τ² = 1.11; p < 0.001), indicating substantial variability beyond sampling error, potentially related to differences in animal species, management practices, geographical settings, bacterial populations, and methodological approaches. Publication bias assessment using funnel plot visualization showed a relatively symmetrical distribution of effect estimates around the overall pooled estimate. Egger’s test did not indicate significant asymmetry (intercept = −0.49; p = 0.46), suggesting no detectable publication bias across the included studies (Fig. 7). Given this high degree of heterogeneity, we carried out subgroup analyses to explore the sources of the variation observed. This approach, stratified in particular by class of antimicrobials, provides a better understanding of the complexity of resistance profiles across the different contexts studied. 

### Subgroup meta-analysis of AMR in food producing animal

The subgroup analysis by antimicrobial class included between 75 and 22,003 tested isolates depending on the antimicrobial category (Table 1). Pooled resistance estimates varied across antimicrobial classes, ranging from 1% to 73%. The highest pooled resistance estimates were observed for sulphonamides (73%; 95% CI: 63–82%; 1,03/1,416) and tetracyclines (69%; 95% CI: 60–76%; 7,99/11,58), followed by penicillin (60%; 95% CI: 48–70%; 9,89/16,48). Regarding other antimicrobial classes, resistance estimates for macrolides and combined folate pathway inhibitors were 55% (95% CI: 36–73%; 2,88/5,24) and 49% (95% CI: 39–59%; 3,206/6,54), respectively. Conversely, lower pooled resistance estimates were observed for lipopeptides (1%; 95% CI: 0–4%), oxazolidinones (3%; 95% CI: 1–9%), glycylcyclines (4%; 95% CI: 1–14%), and carbapenems (10%; 95% CI: 4–21%).Although no evidence of publication bias was observed in the overall meta-analysis, Egger’s test indicated significant asymmetry for fluoroquinolones (*p* = 0.021), macrolides (*p* = 0.008), and penicillin (*p* = 0.00082). These findings suggest that publication bias may affect the estimates for these specific antimicrobial classes and should therefore be interpreted with caution (Table 1).

**Table 1:** Pooled prevalence of antimicrobial resistance

Antimicrobial class	*N*^o^. studies	Total isolates	Pooled prevalence (95% CI)	I² (%)	τ²	Heterogeneity *p*-value	Egger intercept	Egger *p*-value
Aminoglycosides	96	21,106	0.27 (0.21–0.34)	97.2	2.42	< 0.001	−0.89	0.93
Carbapenems	43	5037	0.10 (0.04–0.21)	95.6	8.66	< 0.0001	0.37	0.05
Cephalosporins	80	22,003	0.40 (0.29–0.53)	98.4	5.37	< 0.001	−0.69	0.57
Cephamycins	23	1639	0.14 (0.05–0.31)	88.9	5.93	< 0.0001	−0.41	0.62
Fluoroquinolones	100	19,838	0.30 (0.23–0.37)	97.4	2.60	< 0.001	1.21	**0.02**
Folate pathway inhibitors (combination)	53	6542	0.49 (0.39–0.59)	95.7	2.29	< 0.0001	−0.28	0.41
Folate pathway inhibitors	10	771	0.52 (0.27–0.76)	95.0	2.75	< 0.0001	0.64	0.18
Glycopeptides	14	1274	0.17 (0.04–0.51)	91.9	8.42	< 0.0001	0.51	0.27
Glycylcyclines	6	377	0.04 (0.01–0.14)	73.0	2.43	0.0024	−0.23	0.41
Lincosamides	5	932	0.33 (0.02–0.94)	97.7	14.74	< 0.0001	NR	NR
Lipopeptides	2	166	0.01 (0.00-0.04)	0.0	0.00	0.933	NR	NR
Macrolides	34	5240	0.55 (0.36–0.73)	97.2	4.99	< 0.0001	1.48	**0.008**
Monobactams	15	1,443	0.31 (0.09–0.66)	97.2	7.99	< 0.0001	NR	NR
Nitrofuran	21	2,095	0.26 (0.12–0.47)	95.1	4.02	< 0.0001	−0.62	0.58
Nitroimidazole	2	75	0.52 (0.32–0.71)	67.5	0.238	0.079	NR	NR
Oxazolidinone	4	526	0.03 (0.01–0.09)	66.9	1.29	0.0285	NR	NR
Penicillin–β-lactamase inhibitor	52	6619	0.44 (0.30–0.59)	96.7	4.43	< 0.0001	−0.24	0.83
Penicilin	86	16,486	0.60 (0.48–0.70)	97.3	4.22	< 0.0001	−0.87	**0.000825**
Phenicol	56	5558	0.27 (0.19–0.36)	95.3	2.32	< 0.0001	−0.03	0.30
Phosphonic_acid	2	117	0.29 (0.00–1.00)	95.7	34.23	< 0.0001	NR	NR
Polymyxin	14	1117	0.21 (0.07–0.50)	90.2	5.56	< 0.0001	−0.62	0.58
Quinolone	36	2643	0.44 (0.30–0.58)	93.0	NR	< 0.0001	0.13	0.47
Rifamycin	4	851	0.08 (0.01–0.45)	93.7	4.61	< 0.0001	NR	NR
Tetracycline	82	11,584	0.69 (0.60–0.76)	95.8	2.56	< 0.0001	0.71	0.54
Sulfonamide	18	1,416	0.73 (0.63–0.82)	86.5	0.87	< 0.0001	0.89	0.81
Streptogramin	3	234	0.09 (0.01–0.57)	93.9	4.80	< 0.0001	NR	NR

Data include the number of studies, total isolates, pooled resistance prevalence with 95% confidence intervals (CI), heterogeneity statistics (I² and τ²), the p-value for heterogeneity testing, and Egger’s regression test parameters for assessment of publication bias where applicable

*NR* not reliable (insufficient number of studies to allow valid statistical assessment)

### Correlation between antimicrobials reported as administered in livestock farming systems and antimicrobial resistance profiles reported in laboratory research studies

**Fig. 8:**
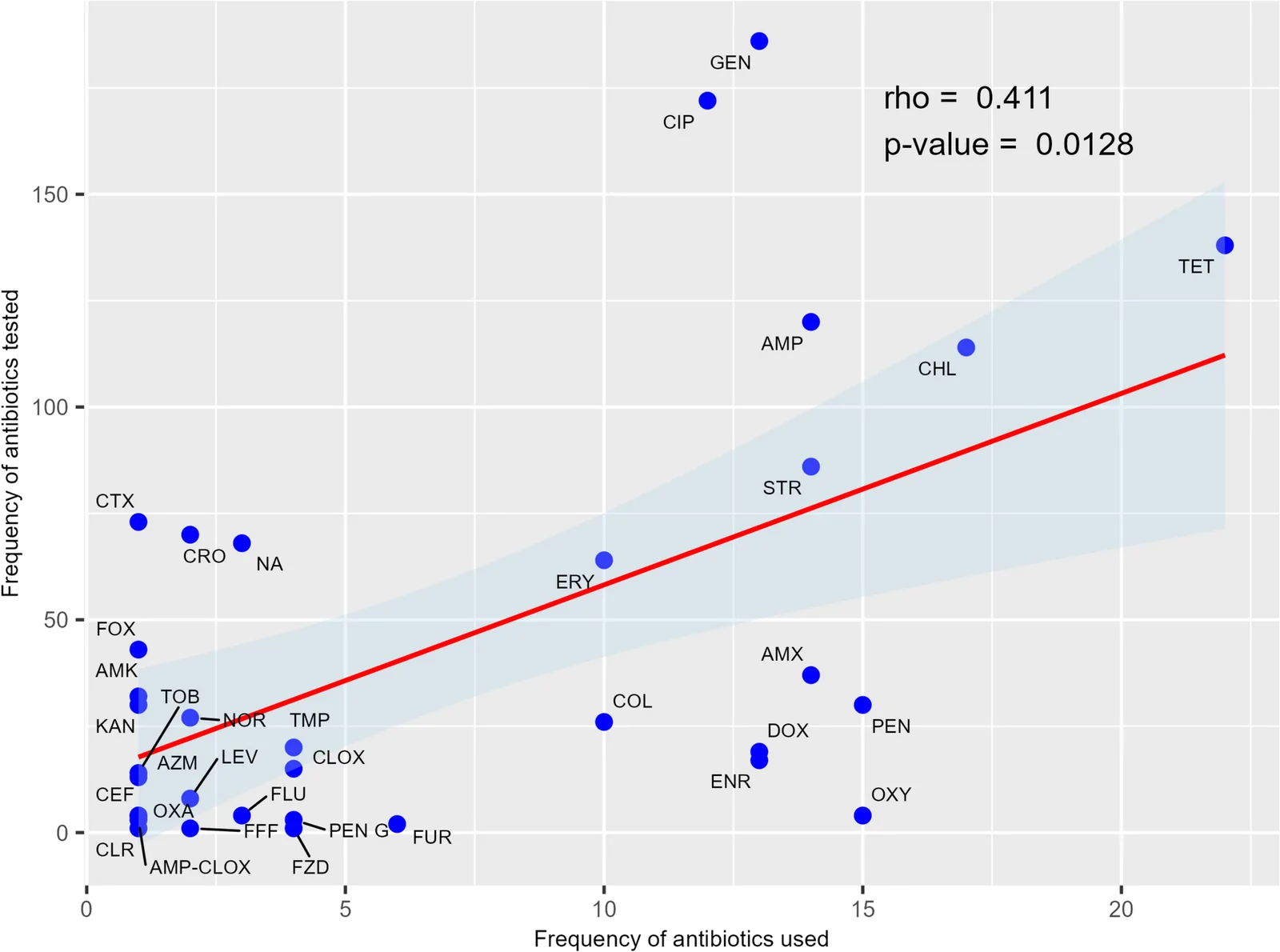
Scatter plot showing the correlation between AMU and AMR. This scatter plot illustrates the relationship between the frequency of reported antimicrobial use and the frequency of resistance reporting. Data are aggregated across all animal species (including terrestrial and aquatic) and all bacterial isolates included in this meta-analysis in West Africa, with each data point representing a specific antimicrobial agent as detailed in Supplementary Figure 1. The trend line represents the Spearman’s rank correlation (rho = 0.411; p = 0.0128), and the shaded area indicates the 95% confidence interval. AMX (Amoxicillin), AMK (Amikacin), GEN (Gentamicin), TOB (Tobramycin), CHL (Chloramphenicol), COL (Colistin), CRO (Ceftriaxon), FOX (Cefoxitin), CIP (Ciprofloxacin), AMP (Ampicillin), ERY (Erythromycin), TET (Tetracycline), STR (Streptomycin), LEV (Levofloxacin), DOX (Doxycycline), FLU (Flumequine), NOR (Norfloxacin), KAN (Kanamycin), TMP (Trimethoprim), CEF (Ceftiofur), PEN (Penicillin), AZM (Azithromycin), FUR (Furazolidone), FFF (Florfenicol), ENR (Enrofloxacin), PEN G (Penicillin G), FZD (Furazolidone), CLOX (Cloxacillin), OXA (Oxacillin), CLR (Clarithromycin), AMP/CLOX (Ampiclox)

The association between reported antimicrobial use and antimicrobial resistance surveillance patterns in food-producing animals was assessed using Spearman’s rank correlation test. A moderate positive correlation was observed between the frequency of antimicrobial use reports and the frequency of resistance reports (ρ = 0.41), with statistical significance (*p* = 0.0128) (Fig. 8). This indicates that antimicrobials frequently reported in livestock use studies tended to be those more frequently investigated in antimicrobial resistance surveillance studies across the region.

The distribution of antimicrobial agents showed distinct patterns. Tetracycline (TET), Ampicillin (AMP), and Chloramphenicol (CHL) were located in the upper-right quadrant, indicating that these antimicrobials were frequently reported in both antimicrobial use and resistance surveillance studies. Conversely, Clindamycin (CLI), Furazolidone (FZD), and Cefotaxime (CTX) were positioned in the lower-left quadrant, reflecting lower reporting frequencies in both domains. Gentamicin (GEN) and Ciprofloxacin (CIP) appeared as outliers, with relatively high resistance surveillance attention despite moderate frequencies of antimicrobial use reporting. The detailed distribution of antimicrobial agents included in this analysis is presented in Supplementary Fig. 1.

## Discussion

In West Africa, the increasing burden of AMR, combined with the often-unregulated use of antimicrobials in livestock production, raises major concerns for public health and food safety [17]. This study summarizes antimicrobial use and antimicrobial resistance profiles in farm animals in West Africa, revealing a significant asymmetry in surveillance management. We observed three distinct patterns: first, a consistency between the use and monitoring of first-line antimicrobials, such as amoxicillin and tetracyclines; second, a proactive monitoring of critical antimicrobials, such as carbapenems, which receive surveillance despite being absent from livestock farming; and third, a critical “blind spot” regarding antimicrobials widely used in veterinary medicine, such as tylosin and tiamulin, which currently lack systematic monitoring. This discrepancy stems from the prioritization of antimicrobials critical to human health, alongside the analytical complexities inherent in monitoring multifaceted field formulations, such as tylodox [18, 19], which complicates the harmonization of test panels. These results confirm a persistent imbalance between the intensive use of certain antimicrobials in livestock farming and their absence from surveillance protocols in west Africa. The disconnect between the intensive use of antimicrobials in west African livestock farming and the inadequacy of surveillance systems stems from a combination of economic pressures and major structural challenges. Whilst the administration of antimicrobials for growth promotion and prophylaxis meets growing food demand, epidemiological monitoring remains hampered by weak regulatory frameworks, the prohibitive cost of testing, a shortage of skilled labour, and the largely informal nature of livestock farming systems. This disconnect, which is common in resource-limited areas, prevents effective monitoring of antimicrobials that actively contribute to the selection and spread of antimicrobial resistance [20, 21].

Tetracycline dominates both antimicrobial use and surveillance parameters, confirming its central role in livestock farming in West Africa. Conversely, increased monitoring of gentamicin and ciprofloxacin, beyond their actual use, highlights a strategy focused on protecting antimicrobials that are critical for human medicine [18]. Conversely, penicillin and oxytetracycline are under-monitored relative to their frequency of use, illustrating a blind spot in veterinary surveillance. Finally, a high density of low-use antimicrobials including CEF (Ceftiofur), FZD (Furazolidone) and CLR (Clarithromycin), remains concentrated at the base of the distribution, illustrating marginal surveillance for less-prescribed agents.

The bacterial diversity observed, with more than 40 different bacteria identified, highlights the complexity of the microbial communities circulating in the livestock systems studied. The predominance of *E. coli* (35.71%) aligns with surveillance data. This prevalence reflects its routine use as a standard indicator of faecal contamination and antimicrobial pressure, as well as its ease of recovery [22, 23]. The high proportion of *Salmonella* spp. can be attributed to the persistence of this bacterium in livestock systems, its ability to survive in the environment, and often limited biosecurity practices, particularly in the poultry and ruminant sectors [24]. The low frequency of other opportunistic enterobacteria, such as *Klebsiella* spp., *Proteus* spp., and *Citrobacter freundii*, is consistent with their opportunistic nature and infrequent status as standard monitoring indicators in livestock production systems. However, their detection is likely maintained by constant environmental transmission from contaminated water and feed sources, coupled with the selective pressure exerted by the non-targeted use of broad-spectrum antimicrobials, which prevents their elimination and sustains their persistence within the farm environment [25]. The limited diversity observed among Gram-positive bacteria, particularly *Enterococcus* spp. and *Staphylococcus aureus*, likely reflects their lower prioritization in veterinary surveillance protocols, despite their significance as reservoirs for antimicrobial resistance, including MR*SA* [26]. Finally, the occasional detection of other major zoonotic pathogens such as *Listeria monocytogenes*, *Campylobacter* spp., and *Yersinia enterocolitica* can be explained by their generally lower prevalence in livestock farming, combined with methodological constraints related to their isolation [27]. Despite their low frequency, their presence remains a concern due to their significant impact on public health and highlights the need for integrated One Health surveillance [27].

The significant variability in antimicrobial use patterns among animal species reflects structural differences in farming systems, health constraints, and disease management strategies. Evermore, variability in AMU patterns across animal species and production systems can be attributed to differences in regulatory and policy frameworks, as well as the implementation of antimicrobial stewardship programmes. Additional contributing factors could include variations in disease burden and endemicity, widespread access to over-the-counter antimicrobials, limited veterinary services and infrastructure/workforce, weak surveillance and diagnostic capacities, and inadequate biosecurity practices. Behavioural and socio-economic factors among livestock producers play a significant role in shaping antimicrobial use practices. These drivers are underpinned by broader structural factors such as system-level conditions encompassing policy environments, infrastructure, social and economic organization, which collectively influence how antimicrobials are accessed, regulated, and utilized within livestock production systems.

Although descriptive differences in antimicrobial use patterns were observed across animal species, post-hoc analyses did not identify statistically significant differences between antimicrobial use purposes within species after Bonferroni correction. These findings suggest that antimicrobial use practices reported in the literature are highly variable across livestock production systems and should therefore be interpreted with caution. The descriptive patterns observed may reflect differences in livestock management practices. The relatively higher proportion of therapeutic antimicrobial use reported in cattle and pigs may be related to their high economic value and to disease management strategies that rely on treatment following the appearance of clinical signs, as previously described in livestock production systems in sub-Saharan Africa and elsewhere [28, 29]. In contrast, the predominance of prophylactic antimicrobial use reported in poultry may reflect preventive flock-level health management practices associated with intensive production systems, high stocking densities, and challenges in implementing effective biosecurity and veterinary oversight. Such practices have been identified as potential drivers of antimicrobial resistance because they increase bacterial exposure to antimicrobials at the population level [30, 31]. Similarly, the predominance of prophylactic use reported in small ruminants and the mixed patterns observed in aquaculture may reflect differences in disease prevention strategies, access to veterinary services, and the availability of standardized treatment protocols [12, 32]. However, these interpretations remain speculative and should be considered in light of the absence of statistically significant differences between antimicrobial use categories.

The low frequency of antimicrobial use for growth promotion observed in this review suggests that antimicrobials are predominantly reported for disease prevention and treatment rather than productivity enhancement. This finding is consistent with previous studies from low- and middle-income countries, where therapeutic and prophylactic applications account for most antimicrobial use in livestock production [33]. Despite a perceived lower therapeutic intensity, small ruminants are major reservoirs of resistant bacteria. The high resistance rates to tetracyclines, penicillin, and macrolides which are often used empirically point to a discrepancy between field observations and self-reported data. These results suggest that prophylactic and zootechnical uses are likely underestimated in farmers’ declarations [34, 35]. Recent studies conducted in West and East Africa have shown high rates of resistance to tetracyclines and beta-lactams in *E. coli*, *Salmonella* spp., and *S. aureus* isolated from livestock, highlighting persistent selection pressure in these sectors [36–38]. The resistance observed to fluoroquinolones in certain bacterial species in small ruminants is particularly worrying, as these antimicrobials are classified as antimicrobials of critical importance for human medicine. Several authors have shown that the emergence of resistance to fluoroquinolones is often linked to a co-selection phenomena involving other antimicrobial class [39–41].

The cephalosporin resistance observed in our study may be associated with the circulation of ESBL-producing bacteria and ESBL-related genes within traditional farming systems, as previously reported [25]. In fish, the resistance profiles observed for penicillin, tetracyclines, macrolides, and sulphonamides confirm the central role of aquaculture in the spread of antimicrobial resistance. Recent reviews have shown that antimicrobial use in aquaculture systems, often for prophylactic purposes and administered via water or feed, promotes prolonged exposure of environmental and enteric bacteria, thereby accelerating the selection of multidrug-resistant strains [42, 43]. Studies conducted in Africa and Asia have reported high levels of resistance to ampicillin, tetracycline, and cotrimoxazole in *E. coli* and *Salmonella* spp. isolated from farmed fish, which is consistent with the findings of our systematic review [42, 44]. The variability in resistance profiles to cephalosporins and fluoroquinolones in fish suggests considerable heterogeneity in aquaculture practices and regulatory frameworks, as also highlighted by Schar et al. [26]. The high burden of antimicrobial resistance observed in food-producing animals has important implications beyond livestock production, as resistant bacteria and antimicrobial resistance genes may circulate between animals, humans, and the environment. Food-producing animals can serve as reservoirs of resistant microorganisms that may reach humans through the consumption of contaminated animal-derived products, occupational exposure among farmers and animal handlers, or environmental dissemination through animal waste and agricultural practices. In addition, mobile genetic elements carrying resistance determinants may facilitate the transfer of antimicrobial resistance genes between bacterial populations across different ecological compartments.

In West Africa, where livestock farming is closely integrated with human communities and environmental resources, these transmission pathways may contribute to the wider dissemination of antimicrobial resistance. Therefore, the resistance patterns identified in livestock should not be considered solely as an animal health concern but as part of a broader One Health challenge. Strengthening integrated surveillance systems that combine antimicrobial use monitoring, animal health data, food chain surveillance, and environmental assessment is essential to better understand and control the spread of antimicrobial resistance across interconnected reservoirs.

The relative preservation of aminoglycoside and carbapenem activity is consistent with their limited or non-existent use in aquaculture and in small ruminants. However, several authors warn against the emergence of resistance to these last-resort antimicrobials via mechanisms of horizontal gene transfer and co-resistance, particularly in heavily contaminated aquatic environments [45].

The global meta-analysis, including 102 studies from livestock in West Africa, revealed a pooled prevalence of antimicrobial resistance of 40% according to the random effects model, with very high heterogeneity. Nevertheless, the very high heterogeneity observed indicates substantial variability among the included studies, reflecting variability in geographical locations, animal production systems, bacterial species, and antimicrobial usage practices. The funnel plot (Fig. 7B) shows a relatively symmetrical distribution, and Egger’s test confirmed the absence of significant publication bias (intercept = − 0.493; *p* = 0.46). The absence of publication bias in the overall analysis does not preclude the presence of bias within specific subgroups. The significant Egger’s test results observed for fluoroquinolones, macrolides, and penicillin may reflect selective reporting, the limited number of studies, or between-study differences within these antimicrobial classes. Consequently, pooled estimates for these classes should be interpreted cautiously. This prevalence is comparable to recent estimates reported in food-producing animals from other emerging regions, such as South Asia and sub-Saharan Africa (38–44%) [46, 47] and similar to those reported in other regions of African regions (35–55%) [2, 48, 49]. However, it is higher than the prevalence observed in several European countries (< 30%) [50, 51]. These regional differences can be explained by stricter regulatory frameworks in Europe, including campaigns to reduce veterinary antimicrobial use and robust surveillance systems, which have led to a decline in consumption since 2011 while still showing high resistance (> 20–50%) in indicator enterobacteria [52]. Analysis of resistance by antimicrobial class revealed the highest prevalence rates for sulphonamides (73%) and tetracyclines (69%). This finding is consistent with their frequent use for prophylactic and therapeutic purposes, their low cost, and their wide availability in extensive and semi-intensive farming systems [53–55]. High resistance to penicillin (60%) and macrolides (55%) also reflects their common use for empirical treatment of bacterial infections in animals [56]. Intermediate prevalence was observed for several classes, including aminoglycosides (27%), fluoroquinolones (30%), cephalosporins (40%), and penicillin-β-lactamase inhibitors (44%). These lower rates, compared to older generations of antimicrobials, are likely linked to more targeted or restricted use, often conditioned by their higher cost or specific regulatory recommendations. Conversely, last-line antimicrobials, such as lipopeptides, oxazolidinones, glycylcyclines, and carbapenems, remain highly effective overall, with their low levels of resistance attributed to very limited or even non-existent use in veterinary medicine [18].

Beyond these specific resistance profiles, this study is of paramount importance for public health and food security in West Africa. It represents one of the first attempts to quantitatively assess the correlation between veterinary antimicrobial use and laboratory-based surveillance laboratory at a regional level.

The observed moderate positive correlation between reported antimicrobial use and resistance surveillance patterns suggests a concordance between antimicrobials frequently described in livestock production systems and those commonly investigated in resistance studies. However, this finding should be interpreted cautiously because antimicrobial use was assessed based on the frequency of reporting in the available literature rather than standardized quantitative consumption indicators, such as treatment frequency, dosage, or biomass-adjusted consumption metrics.

Therefore, the observed relationship represents an ecological association and does not demonstrate a causal link between antimicrobial use and antimicrobial resistance development. Nevertheless, this analysis provides an overview of how antimicrobial use information and resistance surveillance efforts are represented in the current evidence base. The differences observed for some antimicrobials, such as Ciprofloxacin and Gentamicin, may reflect targeted surveillance priorities related to their clinical importance rather than their actual level of use in livestock systems.

These findings highlight the need for integrated surveillance approaches combining quantitative antimicrobial use data with laboratory-based antimicrobial resistance monitoring to better characterize the relationship between antimicrobial exposure and resistance emergence in food-producing animals.

Furthermore, by demonstrating the predominance of therapeutic antimicrobial use, particularly in sectors like poultry and cattle production, this analysis provides essential evidence for the operationalization of National Action Plans (NAPs) against antimicrobial resistance. This high reliance on therapeutic interventions underscores the urgent need to improve biosecurity and farm management to reduce the overall disease burden and subsequent antimicrobial consumption.

### Limitations of the study

This meta-analysis is limited by high heterogeneity (I^2^ = 99.4%) due to the diversity of methods and samples, as well as by the lack of detailed information regarding the specific types and dosages of antimicrobials used in the original studies. Furthermore, the uneven geographical coverage across West Africa limits the overall representativeness of our findings. A critical constraint of this study is the significant disparity in sample sizes between the antimicrobial resistance data (*n* = 140) and the antimicrobial use data (*n* = 41). This imbalance may adversely affect the statistical power and precision of our correlation estimates, thereby constraining the strength of our inferences and the broader conclusions drawn regarding the relationship between veterinary practices and monitored resistance. Another limitation relates to the exclusion of studies reporting both AMU and AMR from the quantitative synthesis. These studies were excluded to ensure the independence of datasets and to avoid potential duplication in the meta-analysis. However, their exclusion may have reduced the amount of information available to explore the ecological association between reported antimicrobial use and antimicrobial resistance. Consequently, the reported correlation should be interpreted as an ecological association based on the available aggregated data and may not fully capture the complexity of the relationship between antimicrobial use and resistance in livestock production systems.

## Conclusion

This systematic review and meta-analysis provide the first regional synthesis of the relationship between AMU and AMR in bacteria isolated from food-producing animals in West Africa. The findings reveal widespread resistance among bacterial isolates, with an overall pooled resistance prevalence of 40%, particularly against sulphonamids, tetracyclines, and penicillin, while carbapenems and other critically important antimicrobials retained relatively low resistance levels. The review also identified important discrepancies between antimicrobials used in livestock production and those included in AMR surveillance, highlighting gaps in current monitoring systems. Furthermore, the moderate positive correlation between reported antimicrobial use and AMR surveillance emphasizes the close relationship between antimicrobial use practices and the occurrence of resistance in bacterial isolates from food-producing animals.

These findings underscore the need to strengthen integrated AMU and AMR surveillance systems across West Africa within a One Health framework, harmonize antimicrobial stewardship policies, and expand resistance monitoring to include antimicrobials widely used in livestock production but currently underrepresented in surveillance programs. Future studies should adopt standardized methodologies and improve geographical coverage to generate more representative regional evidence and support evidence-based interventions aimed at limiting the emergence and spread of antimicrobial-resistant bacteria in food-producing animals.

## Supplementary Information

Supplementary Material 1.

Supplementary Material 2.

Supplementary Material 3.

Supplementary Material 4.

Supplementary Material 5.

## Data Availability

Available upon request.

## Abbreviations

AMR: Antimicrobial Resistance
AMU: Antimicrobial Use
ESBL: Extended-Spectrum Beta-Lactamase
MRSA: Methicillin-Resistant Staphylococcus aureus

## References

[1] Ouedraogo AS, Sanou M, Kissou A, Sanou S, Solaré H, Kaboré F, et al. High prevalence of extended-spectrum ß-lactamase producing enterobacteriaceae among clinical isolates in Burkina Faso. BMC Infect Dis. 2016;16:1–9. 10.1186/s12879-016-1655-3. PMC4939587 27400864

[2] Chilanga F, Kasozi KI, Mazeri S, Paterson GK, Muwonge A A systematic review of antimicrobial resistance transmission inferences at the human-livestock interface in Africa. npj Antimicrob Resist. 2025;3:5:1–11. 10.1038/s44259-025-00126-y. PMC12209416 40588479

[3] French GL. Clinical impact and relevance of antibiotic resistance. Adv Drug Deliv Rev. 2005;57:1514–27. 10.1016/j.addr.2005.04.005. 15978698

[4] Wah K, Tang K, Millar BC, Moore JE, Moore JE Antimicrob Resist (AMR). 2023;80:1–11. 10.3389/bjbs.2023.11387.

[5] Busch G, Kassas B, Palma MA, Risius A Perceptions of antibiotic use in livestock farming in Germany, Italy and the United States. Livest Sci. 2020;241:104251. 10.1016/j.livsci.2020.104251.

[6] Muteeb G, Rehman MT, Shahwan M, Aatif M. Origin of Antibiotics and Antibiotic Resistance, and Their Impacts on Drug Development: A Narrative Review. Pharmaceuticals. 2023;16:1–54. 10.3390/ph16111615. PMC10675245 38004480

[7] Erdaw MM. Contribution, prospects and trends of livestock production in sub-Saharan Africa: a review. Int J Agric Sustain. 2023;21. 10.1080/14735903.2023.2247776.

[8] Food and Agriculture Organization of the United Nations. Averting risks to the food chain. 2017. https://openknowledge.fao.org/server/api/core/bitstreams/966d140d-e5cf-4923-af97-3fd8d90a51fe/content.

[9] Georgakakos CB, Hicks BJ, Walter MT. Farmer perceptions of dairy farm antibiotic use and transport pathways as determinants of contaminant loads to the environment. J Environ Manage. 2021;281:111880. 10.1016/j.jenvman.2020.111880. 33401120

[10] Aworh MK, Kwaga J, Okolocha E, Harden L, Hull D, Hendriksen RS, et al. Extended-spectrum ß-lactamase-producing Escherichia coli among humans, chickens and poultry environments in Abuja, Nigeria. One Heal Outlook. 2020. 10.1186/s42522-020-00014-7. 2. PMC7993457 33829130

[11] Adebowale OO, Adeyemo FA, Bankole N. Farmers ’ Perceptions and Drivers of Antimicrobial Use and Abuse in Commercial Pig Production. Int J Environ Res Public Heal. 2020;17. 10.3390/ijerph17103579. PMC7277550 32443741

[12] Soma D, Diarra FBJ, Bonkoungou IJO, Somda NS. Assessing antibiotic use practices on central Burkina Faso cattle farms and the associated risks to environmental and human health contamination: A pilot study. 2025;11:1–12. 10.14202/IJOH.2025.1-12.

[13] Olowe OA, Adewumi O, Odewale G, Ojurongbe O, Adefioye OJ. Phenotypic and molecular characterisation of extended-spectrum beta-lactamase producing Escherichia coli obtained from animal fecal samples in Ado Ekiti, Nigeria. J Environ Public Health. 2015;2015. 10.1155/2015/497980. PMC4568380 26417371

[14] Soré S, Diarra FBJ, Bonkoungou JI, Sawadogo C, Bationo BG, Ky H, et al. Prevalence and characterization of extended-spectrum β -lactamase-producing Escherichia coli and Klebsiella pneumoniae isolated from poultry in Ouagadougou. Burkina Faso. 2021;15:447–53. 10.5897/AJMR2021.9489.

[15] Page MJ, Mckenzie JE, Bossuyt PM, Boutron I, Hoffmann C, Mulrow CD, et al. The PRISMA 2020 statement: an updated guideline for reporting systematic reviews. Syst reviews Meta-Analyses. 2021. 10.1136/bmj.n71. PMC8008539 33781348

[16] Zachary Munn S, Moola. Karolina Lisy DR and CT. Methodological guidance for systematic reviews of observational epidemiological studies reporting prevalence and cumulative incidence data. 2015;147–53. 10.1097/XEB.0000000000000054 26317388

[17] Aarestrup FM. The livestock reservoir for antimicrobial resistance: a personal view on changing patterns of risks, effects of interventions and the way forward. Phil Trans R Soc B. 2015;370. 10.1098/rstb.2014.0085. PMC4424434 25918442

[18] WHO. WHO List of Medically Important Antimicrobials A risk management tool for mitigating antimicrobial resistance due to non-human use. 2024.

[19] Alabi MA, Lin J. Antibiotic Use in Livestock: A Driver of Resistance in Africa and the Path to Safer Alternatives. 2025. 10.1002/mbo3.70122 PMC12602276 41214790

[20] Aarestrup FM. The livestock reservoir for antimicrobial resistance: a personal view on changing patterns of risks, effects of interventio ns and the way forward. 2015. 10.1098/rstb.2014.0085 PMC4424434 25918442

[21] Boeckel TP, Van, Brower C, Gilbert M, Grenfell BT, Levin SA. Global trends in antimicrobial use in food animals. PNAS. 2015;1–6. 10.1073/pnas.1503141112. PMC4426470 25792457

[22] Boeckel TP, Van, Glennon E, Chen D, Gilbert M, Robinson T, Grenfell B, et al. Reducing antimicrobial use in food animals: Consider user fees and regulatory caps on veterinary use. Science. 2017;351:1350. 10.1126/science.aao1495. PMC6510296 28963240

[23] Mughini-gras L, Dorado-garcía A, Duijkeren E, Van BG, Van Den, Dierikx CM, Bonten MJM. Articles Attributable sources of community-acquired carriage of Escherichia coli containing β-lactam antibiotic resistance genes: a population-based modelling study. 2019;357–69. 10.1016/S2542-5196(19)30130-5. 31439317

[24] Nhung NT, Cuong NV, Thwaites G, Carrique-mas J. Antimicrobial Usage and Antimicrobial Resistance in Animal Production in Southeast Asia. Rev. 2016;1–24. 10.3390/antibiotics5040037. PMC5187518 27827853

[25] Poirel L, Madec J, Lupo A, Schink A, Kieffer N, Nordmann P, et al. Antimicrobial Resistance in Escherichia coli. Microbiol Spectr. 2018;6:1–27. 10.1128/microbiolspec.ARBA-0026-2017.Correspondence. PMC11633601 30003866

[26] Schar D, Klein EY, Laxminarayan R, Gilbert M, Van Boeckel TP. Global trends in antimicrobial use in aquaculture. Sci Rep. 2020;1–9. 10.1038/s41598-020-78849-3. PMC7736322 33318576

[27] WHO. Global Antimicrobial Resistance and Use Surveillance System (GLASS) Report 2022. 2022.

[28] Grace D. Review of Evidence on Antimicrobial Resistance and Animal Agriculture in Developing Countries Delia Grace. 2015. 10.12774/eod_cr.june2015.graced.

[29] Landers TF. A Review of Antibiotic Use in Food Animals: Perspective, Policy, and Potential. 2012; 127. 10.1177/003335491212700103. PMC3234384 22298919

[30] Auger L, Saucier L, Gaucher M, Vandenberg GW, Vincent AT, Thibodeau A, et al. Use, Risk and Revalorization of Veterinary Antibiotics : A Canadian Perspective. Antibiotics. 2025;14:1–25. 10.3390/antibiotics14070665. PMC12291874 40723969

[31] Henriksson PJG, Rico A, Troell M, Klinger DH. Unpacking factors influencing antimicrobial use in global aquaculture and their implication for management: a review from a systems perspective. Sustain Sci. 2018;13:1105–20. 10.1007/s11625-017-0511-8. PMC6086308 30147798

[32] Kimeli P, Mwacalimba K, Tiernan R, Mijten E, Miroshnychenko T, Nautrup BP. Important Diseases of Small Ruminants in Sub-Saharan Africa: A Review with a Focus on Current Strategies for Treatment and Control in Smallholder Systems. Animals. 2025;15:1–32. 10.3390/ani15050706. PMC11898534 40075989

[33] Abate TA, Birhanu AG. Antibiotic Use in Livestock and Environmental Antibiotic Resistance: A Narrative Review. 2025. 10.1177/11786302251357775 PMC12304651 40735169

[34] Bonkoungou I, Ouindgueta, Edith M, Nikiema M, Garba Z, Bako E, et al. Detection of bla CTX-M, bla TEM, and bla SHV genes in ESBL- producing enterobacterales from poultry farms in the peri-urban area of Ouagadougou. Burkina Faso. 2025;17:14–22. 10.5897/JMA2024.0472.

[35] Kagambèga A, Lienemann T, Frye JG, Barro N, Haukka K. Whole genome sequencing of multidrug- resistant Salmonella enterica serovar Typhimurium isolated from humans and poultry in Burkina Faso. Trop Med Health. 2018;46:18–22. 10.1186/s41182-018-0086-9. PMC5808401 29449781

[36] Dagnaw M, Belete A, Wondu B, Lulie S, Admassu B, Zemene M et al. Systematic review and meta-analysis of Staphylococcus aureus at the human-animal-environment Interface in eastern Africa: Prevalence, virulence genes, and antimicrobial resistance profile. 2025;21. 10.1016/j.onehlt.2025.101229. PMC12642166 41292610

[37] Dione MM, Amia WC, Ejobi F, Ouma EA, Wieland B Supply Chain and Delivery of Antimicrobial Drugs in Smallholder Livestock Production Systems in Uganda. 2021;8:1–13. 10.3389/fvets.2021.611076. PMC8455991 34568469

[38] Soma D, Juste I, Bonkoungou O, Garba Z, Bintou F, Diarra J, et al. Extended-Spectrum Beta-Lactamase-Producing and Multidrug-Resistant Escherichia coli and Klebsiella spp. from the Human – Animal – Environment Interface on Cattle Farms in Burkina Faso. Microbiol Res. 2024;2015:2286–97. 10.3390/microbiolres15040153.

[39] Aarestrup FM, Wegener HC. The effects of antibiotic usage in food animals on the development of antimicrobial resistance of importance for humans in Campylobacter and Escherichia coli. Microbes Infect. 1999;1999:639–44. 10.1016/s1286-4579(99)80064-1 10611741

[40] Zhang L, Ye M, Dong Y, Yuan L, Xiang J, Yu X. Resistance Mechanisms of Fluoroquinolone in Escherichia coli Isolated from Taihe Black-Boned Silky Fowl Exhibiting Abnormally Slow Fluoroquinolone Metabolism in Jiangxi, China. Antibiotics. 2025;14:1–15. 10.3390/antibiotics14090955. PMC12466724 41009933

[41] Callens B, Cargnel M, Sarrazin S, Dewulf J, Hoet B, Vermeersch K, et al. Associations between a decreased veterinary antimicrobial use and resistance in commensal Escherichia coli from Belgian livestock species (2011–2015). Prev Vet Med. 2018;157:50–8. 10.1016/j.prevetmed.2017.10.013. 30086849

[42] Okeke ES, Chukwudozie KI, Nyaruaba R, Ita RE. Antibiotic resistance in aquaculture and aquatic organisms: a review of current nanotechnology applications for sustainable management. Springer Berlin Heidelberg; 2022. 10.1007/s11356-022-22319-y. PMC9376131 35969340

[43] Ali E, Mohammed H, Kovács B, Kuunya R, Omaima E, Mustafa A, et al. Antibiotic Resistance in Aquaculture: Challenges, Trends Analysis, and Alternative Approaches. Antibiotics. 2025;14:1–26. 10.3390/antibiotics14060598. PMC12189707 40558188

[44] Okon EM, Okocha RC, Adesina BT, Ehigie JO, Alabi OO, Bolanle AM, South Africa. Antimicrobial resistance in fi sh and poultry: Public health implications for animal source food production in Nigeria, Egypt, and. 2022;1–19. 10.3389/frabi.2022.1043302. PMC11732016 39816413

[45] Cacace D, Fatta-kassinos D, Manaia CM, Cytryn E, Kreuzinger N, Rizzo L, et al. Antibiotic resistance genes in treated wastewater and in the receiving water bodies: A pan-European survey of urban settings. Water Res. 2019;162:320–30. 10.1016/j.watres.2019.06.039. 31288142

[46] Uzzaman N, Asaduzzaman M. Prevalence, distribution and antimicrobial resistance profiles in poultry meat samples from India : a systematic review. 2025; 1–14. 10.3389/fvets.2025.1672628. PMC12616860 41246263

[47] Olaitan MO, Orababa OQ, Shittu RB, Oyediran AA, Obunukwu GM, Arowolo MT, et al. Extended-spectrum beta-lactam-resistant Klebsiella pneumoniae in sub-Saharan Africa: a systematic review and meta-analysis from a One Health perspective. BMC Infect Dis. 2025;25. 10.1186/s12879-025-11276-9. PMC12210512 40597808

[48] Donkor ES, Odoom A, Osman A, Darkwah S, Kotey FCN. A Systematic Review on Antimicrobial Resistance in Ghana from a One Health Perspective. Antibiotics. 2024;16. 10.3390/antibiotics13070662. PMC11274323 39061344

[49] Somda NS, Tankoano A, Kaboré D, Bonkoungou JOI, Kpoda DS, Dabiré Y, et al. A Systematic Review and Meta-analysis of Antibiotic Resistance of Foodborne Pathogenic Bacteria in West Africa Between 2010 and 2020. J Food Prot. 2023;86:100061. 10.1016/j.jfp.2023.100061. 36916564

[50] Morar PR, Ban-cucerzan A, Popa A. Systematic Review of the Occurrence and Antimicrobial Resistance Profile of Foodborne Pathogens from Enterobacteriaceae in Wild Ungulates Within the European Countries. Pathogens. 2024;13. 10.3390/pathogens13121046. PMC11728525 39770307

[51] 2025; ECDC. The European Union summary report on antimicrobial resistance in zoonotic and indicator bacteria from humans, animals and food in 2022–2023, 10.2903/j.efsa.2025.9237. PMC10900121 38419967

[52] ECDC. Antimicrobial Resistance in the EU/EEA: A One Health Response. 2022. https://www.ecdc.europa.eu/sites/default/files/documents/antimicrobial-resistance-policy-brief-2022.pdf.

[53] Abdulkadir A, Ajayi MO, Kusfa HA. Surveillance of Antimicrobial Use in Animal Production: A Cross-Sectional Study of Kaduna Metropolis, Nigeria. 2025. 10.3390/msf2025035004.

[54] Aworh MK, Ekeng E, Nilsson P, Egyir B, Owusu-Nyantakyi C, Hendriksen RS. Extended-Spectrum ß-Lactamase-Producing Escherichia coli Among Humans, Beef Cattle, and Abattoir Environments in Nigeria. Front Cell Infect Microbiol. 2022;12:1–11. 10.3389/fcimb.2022.869314. PMC9021871 35463650

[55] Adebowale OO, Jimoh AB, Adebayo OO, Alamu AA, Adeleye AI, Fasanmi OG, et al. Evaluation of antimicrobial usage in companion animals at a Veterinary Teaching Hospital in Nigeria. Sci Rep. 2023;1–16. 10.1038/s41598-023-44485-w. PMC10598005 37875528

[56] Sithole V, Amoako DG, Luther A, Abia K, Perrett K, Bester LA, et al. Characterization of Campylobacter spp. in Intensive Pig Production in South Africa. Pathogens. 2021;10. 10.3390/pathogens10040439. PMC8067824 33917115

