# Supplementary material for: Association between antimicrobials administered and antimicrobial resistance profiles in food-producing animals in West Africa: a systematic review and meta-analysis
Source: BMC Vet Res. 2026 Oct 4;22:653. doi: 10.1186/s12917-026-05971-1 (PMC13729510; doi:10.1186/s12917-026-05971-1)
Supplement: Supplementary file 2 [file 12917_2026_5971_MOESM4_ESM.docx]

**Supplementary Tables**

**Table 1:** Search Strategy

| **DATABASE** | **Search field** | **Boolean Search Phrase** | **Final Records Retrieved** |
| --- | --- | --- | --- |
| PubMed | Default search field | (("antibiotic use" OR "antimicrobial use"OR "antibiotic consumption" OR "antimicrobial resistance"OR "antibiotic resistance"OR "AMR") AND ("livestock" OR "cattle" OR "poultry" OR "fish"OR "pig" OR “goat” OR “sheep” OR "animal farming" OR "food animals") AND ("Benin" OR "Burkina Faso" OR "Cabo Verde" OR "Côte d'Ivoire" OR "Ivory Coast" OR "Cote d’Ivoire" OR "Gambia" OR "Ghana" OR "Guinea" OR "Guinea-Bissau" OR "Liberia" OR "Mali" OR "Niger" OR "Nigeria" OR "Senegal" OR "Sierra Leone" OR "Togo")) | 224 |
| SCOPUS | Default search field | ("antibiotic use" OR "antimicrobial use" OR "antibiotic consumption") AND ("antimicrobial resistance" OR "antibiotic resistance" OR "AMR") AND ("livestock" OR "cattle" OR "poultry" OR "pig" OR "fish" OR “goat” OR “sheep” OR "animal farming" OR "food animals") AND ("West Africa" OR "Benin" OR "Burkina Faso" OR "Cabo Verde" OR "Côte d'Ivoire" OR "Ivory Coast" OR "Cote d’Ivoire" OR "Gambia" OR "Ghana" OR "Guinea" OR "Guinea-Bissau" OR "Liberia" OR "Mali" OR "Niger" OR "Nigeria" OR "Senegal" OR "Sierra Leone" OR "Togo") | 320 |
| AJOL (African Journals Online) | Default search field | ("antibiotic use" OR "antimicrobial use" OR "antibiotic consumption") AND ("antimicrobial resistance" OR "antibiotic resistance" OR "AMR") AND ("livestock" OR "cattle" OR "poultry" OR "pig" OR "fish" OR “goat” OR “sheep” OR "animal farming" OR "food animals") AND ("West Africa" OR "Benin" OR "Burkina Faso" OR "Cabo Verde" OR "Côte d'Ivoire" OR "Ivory Coast" OR "Cote d’Ivoire" OR "Gambia" OR "Ghana" OR "Guinea" OR "Guinea-Bissau" OR "Liberia" OR "Mali" OR "Niger" OR "Nigeria" OR "Senegal" OR "Sierra Leone" OR "Togo") | 261 |
| Google scholar | General search interface | ("antibiotic use" OR "antimicrobial use" OR "antibiotic consumption") AND ("antimicrobial resistance" OR "antibiotic resistance" OR "AMR") AND ("animal farming" OR "livestock" OR "cattle" OR "poultry" OR "pig" OR "fish" OR “goat” OR “sheep” OR "food animals") AND ("West Africa" OR "Benin" OR "Burkina Faso" OR "Cabo Verde" OR "Côte d'Ivoire" OR "Ivory Coast" OR "Gambia" OR "Ghana" OR "Guinea" OR "Guinea-Bissau" OR "Liberia" OR "Mali" OR "Niger" OR "Nigeria" OR "Senegal" OR "Sierra Leone" OR "Togo") | 360 |

**Table 2.** Statistical summary of antimicrobial use prevalence and pairwise comparisons by motive and species.

| **Animals_species** | **Comparison** | **Z** | **P.unadj** | **P.adj** |
| --- | --- | --- | --- | --- |
| cattle | Growth - Prophylaxis | 1.354478265 | 0.175583843 | 0.263375765 |
| cattle | Growth - Therapeutic | 1.408657396 | 0.158936498 | 0.476809495 |
| cattle | Prophylaxis - Therapeutic | 0.054179131 | 0.956792448 | 0.956792448 |
| fish | Growth - Prophylaxis | 1.062170009 | 0.288158512 | 0.864475537 |
| fish | Growth - Therapeutic | 0.849736007 | 0.395471875 | 0.593207812 |
| fish | Prophylaxis - Therapeutic | -0.212434002 | 0.831768461 | 0.831768461 |
| goat | Growth - Prophylaxis | 0.071083441 | 0.943331347 | 0.943331347 |
| goat | Growth - Therapeutic | 0.382073493 | 0.702406852 | 1 |
| goat | Prophylaxis - Therapeutic | 0.310990053 | 0.755808184 | 1 |
| pig | Growth - Prophylaxis | -0.077444906 | 0.938269618 | 0.938269618 |
| pig | Growth - Therapeutic | 1.153636861 | 0.24864908 | 0.372973619 |
| pig | Prophylaxis - Therapeutic | 1.231081767 | 0.218292284 | 0.654876852 |
| poultry | Growth - Prophylaxis | -1.458207762 | 0.144783285 | 0.217174927 |
| poultry | Growth - Therapeutic | -1.50712081 | 0.131779694 | 0.395339081 |
| poultry | Prophylaxis - Therapeutic | -0.048913048 | 0.960988591 | 0.960988591 |
| sheep | Growth - Prophylaxis | 0.071083441 | 0.943331347 | 0.943331347 |
| sheep | Growth - Therapeutic | 0.382073493 | 0.702406852 | 1 |
| sheep | Prophylaxis - Therapeutic | 0.310990053 | 0.755808184 | 1 |

**Legend:** This table presents the median prevalence rates and interquartile ranges (IQR) for each usage motive disaggregated by animal species. The p-values shown are derived from the Kruskal-Wallis test for global comparisons between motives within each species, followed by Dunn’s post-hoc test with Benjamini-Hochberg correction for pairwise comparisons. Results indicate a significant predominance of therapeutic and prophylactic motives over growth promotion across all terrestrial livestock sectors (p < 0.05).

**Table 3:** Distribution of pathogen isolated

| **Pathogen** | **Total number of pathogen(n)** | **Relative abundance (%) *** |
| --- | --- | --- |
| *Campylobacter* | 1 | 0.55 |
| *Campylobacter coli* | 2 | 1.10 |
| *Campylobacter jejuni* | 2 | 1.10 |
| *Campylobacter spp* | 1 | 0.55 |
| *Citrobacter freundii* | 2 | 1.10 |
| *Enterobacter aerogenes* | 1 | 0.55 |
| *Enterobacter cloacae* | 1 | 0.55 |
| *Enterobacter spp* | 1 | 0.55 |
| *Enterococcus casseliflavus* | 1 | 0.55 |
| *Enterococcus durans* | 2 | 1.10 |
| *Enterococcus faecalis* | 2 | 1.10 |
| *Enterococcus faecium* | 2 | 1.10 |
| *Enterococcus galinarum* | 1 | 0.55 |
| *Enterococcus hirae* | 2 | 1.10 |
| *Enterococcus innesii* | 1 | 0.55 |
| *Enterococcus lactis* | 1 | 0.55 |
| *Enterococcus pseudoavium* | 1 | 0.55 |
| *Enterococcus spp* | 4 | 2.20 |
| *Enterococcus thailandicus* | 1 | 0.55 |
| *Escherichia coli* | 65 | 35.71 |
| *Klebsiella pneumoniae* | 6 | 3.30 |
| *Klebsiella spp* | 8 | 4.40 |
| *Listeria monocytogenes* | 1 | 0.55 |
| MRSA | 5 | 2.75 |
| *Mammaliicoccus sciuri* | 1 | 0.55 |
| *Proteus mirabilis* | 2 | 1.10 |
| *Proteus spp* | 1 | 0.55 |
| *Proteus vulgaris* | 2 | 1.10 |
| *Providencia spp* | 1 | 0.55 |
| *Pseudomonas aeruginosa* | 2 | 1.10 |
| *Pseudomonas spp* | 1 | 0.55 |
| *Salmonella enterica* | 3 | 1.65 |
| *Salmonella* Enteritidis | 1 | 0.55 |
| *Salmonella* Gallinarum | 1 | 0.55 |
| *Non-typhoidal Salmonella* | 1 | 0.55 |
| *Salmonella spp* | 29 | 15.93 |
| *Salmonella* Typhi | 4 | 2.20 |
| *Shigella spp* | 2 | 1.10 |
| *Staphylococcus arlettae* | 1 | 0.55 |
| *Staphylococcus aureus* | 5 | 2.75 |
| *Staphylococcus chromogenes* | 1 | 0.55 |
| *Staphylococcus epidermidis* | 2 | 1.10 |
| *Staphylococcus haemolyticus* | 2 | 1.10 |
| *Staphylococcus piscifermentans* | 1 | 0.55 |
| *Staphylococcus pseudintermedius* | 1 | 0.55 |
| *Staphylococcus saprophyticus* | 1 | 0.55 |
| *Staphylococcus simulans* | 1 | 0.55 |
| *Staphylococcus xylosus* | 1 | 0.55 |
| *Yersinia enterocolitica* | 1 | 0.55 |
| *TOTAL* | 182 | 100 |

**Legend:** **Percentages (Relative abundance) are calculated based on the total pool of bacterial isolates (N = 182) recovered from the 140 studies included on antimicrobial resistance studies.*

**Table 4:** Joanna Briggs Institute (JBI) quality assessment of studies included in the quantitative meta-analysis (102 AMR studies and 21 AMU studies)

|  |  | **Joanna Briggs Institute’s critical appraisal questions** | | | | | | | | | | |  |  |  |  |
| --- | --- | --- | --- | --- | --- | --- | --- | --- | --- | --- | --- | --- | --- | --- | --- | --- |
| **Author and Year** | **Total Sample Size** | **Q1** | **Q2** | **Q3** | **Q4** | **Q5** | **Q6** | **Q7** | **Q8** | **Q9** | **Score** | **% Yes** | **% No** | **% Unclear** | **Stufy quality** | **Overall appraisal** |
| Dougnon 2023 [1] | 180 | Y | Y | Y | Y | Y | Y | Y | Y | Y | 9 | 100% | 0% | 0% | High | Include |
| Fashae 2021 [2] | 250 | Y | Y | Y | Y | Y | Y | Y | Y | Y | 9 | 100% | 0% | 0% | High | Include |
| Abeni 2016 [3] | 400 | Y | Y | Y | Y | Y | Y | Y | Y | Y | 9 | 100% | 0% | 0% | High | Include |
| Abubakar 2016 [4] | 246 | Y | Y | Y | Y | Y | Y | Y | Y | Y | 9 | 100% | 0% | 0% | High | Include |
| Abubakar 2025 [5] | 450 | Y | Y | Y | Y | Y | Y | Y | Y | Y | 9 | 100% | 0% | 0% | High | Include |
| Adebayo 2021 [6] | 726 | Y | Y | Y | Y | Y | Y | Y | Y | Y | 9 | 100% | 0% | 0% | High | Include |
| Adebowale 2022 [7] | 200 | Y | Y | Y | Y | Y | Y | Y | Y | Y | 9 | 100% | 0% | 0% | High | Include |
| Adeluwoye-Ajayi 2021 [8] | 9 | N | Y | N | Y | Y | Y | Y | Y | Y | 7 | 78% | 22% | 0% | High | Include |
| Adenipekun 2015 [9] | 600 | Y | Y | Y | Y | Y | Y | Y | Y | Y | 9 | 100% | 0% | 0% | High | Include |
| Agbankpe 2022 [10] | 248 | Y | Y | Y | Y | Y | Y | Y | Y | Y | 9 | 100% | 0% | 0% | High | Include |
| Agusi 2024 [11] | 179 | Y | Y | Y | Y | Y | Y | Y | Y | Y | 9 | 100% | 0% | 0% | High | Include |
| Ahmad 2023 [12] | 354 | Y | Y | Y | Y | Y | Y | Y | Y | Y | 9 | 100% | 0% | 0% | High | Include |
| Ajayi 2019 [13] | 9 | N | Y | N | Y | Y | Y | Y | Y | Y | 7 | 78% | 22% | 0% | High | Include |
| Ajayi 2023 [14] | 9 | N | Y | N | Y | Y | Y | Y | Y | Y | 7 | 78% | 22% | 0% | High | Include |
| Ajibola 2025 [15] | 168 | Y | Y | Y | Y | Y | Y | Y | Y | Y | 9 | 100% | 0% | 0% | High | Include |
| Akanbi 2022 [16] | 627 | Y | Y | Y | Y | Y | Y | Y | Y | Y | 9 | 100% | 0% | 0% | High | Include |
| Akeem 2020 [17] | 315 | Y | Y | Y | Y | Y | Y | Y | Y | Y | 9 | 100% | 0% | 0% | High | Include |
| Akeem 2025 [18] | 200 | Y | Y | Y | Y | Y | Y | Y | Y | Y | 9 | 100% | 0% | 0% | High | Include |
| Akre 2022 [19] | 35 | N | Y | N | Y | Y | Y | Y | Y | Y | 7 | 78% | 22% | 0% | High | Include |
| Amaechi 2015 [20] | 720 | Y | Y | Y | Y | Y | Y | Y | Y | Y | 9 | 100% | 0% | 0% | High | Include |
| Anyanwu 2022 [21] | 388 | Y | Y | Y | Y | Y | Y | Y | Y | Y | 9 | 100% | 0% | 0% | High | Include |
| Ariom 2025 [22] | 100 | Y | Y | Y | Y | Y | Y | Y | Y | Y | 9 | 100% | 0% | 0% | High | Include |
| Assoumy 2021 [23] | 231 | Y | Y | Y | Y | Y | Y | Y | Y | Y | 9 | 100% | 0% | 0% | High | Include |
| Aworh 2020 [24] | 111 | Y | Y | Y | Y | Y | Y | Y | Y | Y | 9 | 100% | 0% | 0% | High | Include |
| Aworh 2022 [25] | 448 | Y | Y | Y | Y | Y | Y | Y | Y | Y | 9 | 100% | 0% | 0% | High | Include |
| Ayeni 2015 [26] | 45 | N | Y | N | Y | Y | Y | Y | Y | Y | 7 | 78% | 22% | 0% | High | Include |
| Bamidele 2022 [27] | 120 | Y | Y | Y | Y | Y | Y | Y | Y | Y | 9 | 100% | 0% | 0% | High | Include |
| Bedekelabou 2020 [28] | 117 | Y | Y | Y | Y | Y | Y | Y | Y | Y | 9 | 100% | 0% | 0% | High | Include |
| Beshiru 2024 [29] | 400 | Y | Y | Y | Y | Y | Y | Y | Y | Y | 9 | 100% | 0% | 0% | High | Include |
| Bonkoungou 2025 [30] | 37 | N | Y | Y | N | Y | Y | Y | Y | Y | 7 | 78% | 22% | 0% | High | Include |
| Bouda 2019 [31] | 280 | Y | Y | Y | Y | Y | Y | Y | Y | Y | 9 | 100% | 0% | 0% | High | Include |
| Chika 2017 [32] | 40 | N | Y | N | Y | Y | Y | Y | Y | Y | 7 | 78% | 22% | 0% | High | Include |
| Chika 2018 [33] | 120 | Y | Y | Y | Y | Y | Y | Y | Y | Y | 9 | 100% | 0% | 0% | High | Include |
| Coulibaly 2019 [34] | 420 | Y | Y | Y | Y | Y | Y | Y | Y | Y | 9 | 100% | 0% | 0% | High | Include |
| Doubra 2025 [35] | 100 | Y | Y | Y | Y | Y | Y | Y | Y | Y | 9 | 100% | 0% | 0% | High | Include |
| Egbule 2019 [36] | 200 | Y | Y | Y | Y | Y | Y | Y | Y | Y | 9 | 100% | 0% | 0% | High | Include |
| Ejeh 2017 [37] | 105 | Y | Y | Y | Y | Y | Y | Y | Y | Y | 9 | 100% | 0% | 0% | High | Include |
| Ejikeugwu 2016 [38] | 50 | N | Y | N | Y | Y | Y | Y | Y | Y | 7 | 78% | 22% | 0% | High | Include |
| Ejikeugwu 2021 [39] | 240 | Y | Y | Y | Y | Y | Y | Y | Y | Y | 9 | 100% | 0% | 0% | High | Include |
| Eyram 2017 [40] | 24 | N | Y | N | Y | Y | Y | Y | Y | Y | 7 | 78% | 22% | 0% | High | Include |
| Fagbamila 2023 [41] | 2615 | Y | Y | Y | Y | Y | Y | Y | Y | Y | 9 | 100% | 0% | 0% | High | Include |
| Fashae 2017 [42] | 422 | Y | Y | Y | Y | Y | Y | Y | Y | Y | 9 | 100% | 0% | 0% | High | Include |
| Gaddafi 2021 [43] | 212 | Y | Y | Y | Y | Y | Y | Y | Y | Y | 9 | 100% | 0% | 0% | High | Include |
| Gaddafi 2022 [44] | 200 | Y | Y | Y | Y | Y | Y | Y | Y | Y | 9 | 100% | 0% | 0% | High | Include |
| Gbanamou 2024 [45] | 100 | Y | Y | Y | Y | Y | Y | Y | Y | Y | 9 | 100% | 0% | 0% | High | Include |
| Gbonon 2018 [46] | 71 | Y | Y | Y | Y | Y | Y | Y | Y | Y | 9 | 100% | 0% | 0% | High | Include |
| Gladys 2023 [47] | 370 | Y | Y | Y | Y | Y | Y | Y | Y | Y | 9 | 100% | 0% | 0% | High | Include |
| Ibrahim 2019 [48] | 1500 | Y | Y | Y | Y | Y | Y | Y | Y | Y | 9 | 100% | 0% | 0% | High | Include |
| Igbinosa 2020 [49] | 81 | Y | Y | Y | Y | Y | Y | Y | Y | Y | 9 | 100% | 0% | 0% | High | Include |
| Iliya 2019 [50] | 120 | Y | Y | Y | Y | Y | Y | Y | Y | Y | 9 | 100% | 0% | 0% | High | Include |
| Isichei-Ukah 2024 [51] | 20 | N | Y | N | Y | Y | Y | Y | Y | Y | 7 | 78% | 22% | 0% | High | Include |
| Isoken 2020 [49] | 81 | Y | Y | Y | Y | Y | Y | Y | Y | Y | 9 | 100% | 0% | 0% | High | Include |
| Jajere 2015 | 154 | Y | Y | Y | Y | Y | Y | Y | Y | Y | 9 | 100% | 0% | 0% | High | Include |
| Jibril 2021 [52] | 300 | Y | Y | Y | Y | Y | Y | Y | Y | Y | 9 | 100% | 0% | 0% | High | Include |
| Karikari 2017 [53] | 475 | Y | Y | Y | Y | Y | Y | Y | Y | Y | 9 | 100% | 0% | 0% | High | Include |
| Kolapoi 2022 [54] | 186 | Y | Y | Y | Y | Y | Y | Y | Y | Y | 9 | 100% | 0% | 0% | High | Include |
| Kouadio 2017 [55] | 30 | N | Y | N | Y | Y | Y | Y | Y | Y | 7 | 78% | 22% | 0% | High | Include |
| Kyrian-Ogbonna 2021 [56] | 80 | Y | Y | Y | Y | Y | Y | Y | Y | Y | 9 | 100% | 0% | 0% | High | Include |
| Larbi 2021 [57] | 25 | N | Y | N | Y | Y | Y | Y | Y | Y | 7 | 78% | 22% | 0% | High | Include |
| Larbi 2022 [58] | 200 | Y | Y | Y | Y | Y | Y | Y | Y | Y | 9 | 100% | 0% | 0% | High | Include |
| Mansaray 2022 [59] | 100 | Y | Y | Y | Y | Y | Y | Y | Y | Y | 9 | 100% | 0% | 0% | High | Include |
| Matilda 2025 [60] | 20 | N | Y | N | Y | Y | Y | Y | Y | Y | 7 | 78% | 22% | 0% | High | Include |
| Nancy 2024 [61] | 1108 | Y | Y | Y | Y | Y | Y | Y | Y | Y | 9 | 100% | 0% | 0% | High | Include |
| Ngbede 2016 [62] | 260 | Y | Y | Y | Y | Y | Y | Y | Y | Y | 9 | 100% | 0% | 0% | High | Include |
| Njoga 2025 [63] | 400 | Y | Y | Y | Y | Y | Y | Y | Y | Y | 9 | 100% | 0% | 0% | High | Include |
| Obianuju 2019 [64] | 75 | N | Y | N | Y | Y | Y | Y | Y | Y | 7 | 78% | 22% | 0% | High | Include |
| Ogefere 2017 [65] | 120 | Y | Y | Y | Y | Y | Y | Y | Y | Y | 9 | 100% | 0% | 0% | High | Include |
| Okoye 2025 [66] | 180 | Y | Y | Y | Y | Y | Y | Y | Y | Y | 9 | 100% | 0% | 0% | High | Include |
| Olamilekan 2025 [67] | 60 | N | Y | N | Y | Y | Y | Y | Y | Y | 7 | 78% | 22% | 0% | High | Include |
| Oloso 2019 [68] | 1135 | Y | Y | Y | Y | Y | Y | Y | Y | Y | 9 | 100% | 0% | 0% | High | Include |
| Olu-Taiwo 2025 [69] | 300 | Y | Y | Y | Y | Y | Y | Y | Y | Y | 9 | 100% | 0% | 0% | High | Include |
| Olufunke 2022 [70] | 100 | Y | Y | Y | Y | Y | Y | Y | Y | Y | 9 | 100% | 0% | 0% | High | Include |
| Olusola 2025 [71] | 200 | Y | Y | Y | Y | Y | Y | Y | Y | Y | 9 | 100% | 0% | 0% | High | Include |
| Omoshaba 2020 [72] | 200 | Y | Y | Y | Y | Y | Y | Y | Y | Y | 9 | 100% | 0% | 0% | High | Include |
| Osamamwande 2024 [73] | 400 | Y | Y | Y | Y | Y | Y | Y | Y | Y | 9 | 100% | 0% | 0% | High | Include |
| Paintsil 2023 [74] | 1585 | Y | Y | Y | Y | Y | Y | Y | Y | Y | 9 | 100% | 0% | 0% | High | Include |
| Raji 2021 [75] | 500 | Y | Y | Y | Y | Y | Y | Y | Y | Y | 9 | 100% | 0% | 0% | High | Include |
| Raufu 2021 [75] | 1500 | Y | Y | Y | Y | Y | Y | Y | Y | Y | 9 | 100% | 0% | 0% | High | Include |
| Sanou 2022 [76] | 898 | Y | Y | Y | Y | Y | Y | Y | Y | Y | 9 | 100% | 0% | 0% | High | Include |
| Sekyere 2015 [77] | 108 | Y | Y | Y | Y | Y | Y | Y | Y | Y | 9 | 100% | 0% | 0% | High | Include |
| Sidibe 2018 [78] | 515 | Y | Y | Y | Y | Y | Y | Y | Y | Y | 9 | 100% | 0% | 0% | High | Include |
| Soma 2024 [79] | 68 | N | Y | N | Y | Y | Y | Y | Y | Y | 7 | 78% | 22% | 0% | High | Include |
| Sore 2022 [80] | 375 | Y | Y | Y | Y | Y | Y | Y | Y | Y | 9 | 100% | 0% | 0% | High | Include |
| Tahou 2025 [81] | 64 | N | Y | N | Y | Y | Y | Y | Y | Y | 7 | 78% | 22% | 0% | High | Include |
| Tama 2021 [82] | 90 | Y | Y | Y | Y | Y | Y | Y | Y | Y | 9 | 100% | 0% | 0% | High | Include |
| Terese 2022 [83] | 360 | Y | Y | Y | Y | Y | Y | Y | Y | Y | 9 | 100% | 0% | 0% | High | Include |
| Ugbo 2023 [84] | 180 | Y | Y | Y | Y | Y | Y | Y | Y | Y | 9 | 100% | 0% | 0% | High | Include |
| Ugbo 2025 [85] | 75 | N | Y | N | Y | Y | Y | Y | Y | Y | 7 | 78% | 22% | 0% | High | Include |
| Ugwu 2018 [86] | 120 | Y | Y | Y | Y | Y | Y | Y | Y | Y | 9 | 100% | 0% | 0% | High | Include |
| Ugwu 2023 [87] | 107 | Y | Y | Y | Y | Y | Y | Y | Y | Y | 9 | 100% | 0% | 0% | High | Include |
| Vounba 2018 [88] | 58 | N | Y | N | Y | Y | Y | Y | Y | Y | 7 | 78% | 22% | 0% | High | Include |
| Vounba 2019 [89] | 32 | N | Y | N | Y | Y | Y | Y | Y | Y | 7 | 78% | 22% | 0% | High | Include |
| Yao 2018 [90] | 420 | Y | Y | Y | Y | Y | Y | Y | Y | Y | 9 | 100% | 0% | 0% | High | Include |
| Yhiler 2019 [91] | 55 | N | Y | N | Y | Y | Y | Y | Y | Y | 7 | 78% | 22% | 0% | High | Include |
| Yusuf 2023 [92] | 289 | Y | Y | Y | Y | Y | Y | Y | Y | Y | 9 | 100% | 0% | 0% | High | Include |
| Madera 2021 [93] | 50 | Y | Y | Y | Y | Y | Y | Y | Y | Y | 9 | 100% | 0% | 0% | High | Include |
| Gibernau 2025 [94] | 81 | Y | Y | Y | Y | Y | Y | Y | Y | Y | 9 | 100% | 0% | 0% | High | Include |
| Sawadogo 2023 [95] | 100 | Y | Y | Y | Y | Y | Y | Y | Y | Y | 9 | 100% | 0% | 0% | High | Include |
| Alhaji 2021 [96] | 44 | Y | Y | Y | Y | Y | Y | Y | Y | Y | 9 | 100% | 0% | 0% | High | Include |
| Adelowo 2019 [97] | 88 | Y | Y | Y | Y | Y | Y | Y | Y | Y | 9 | 100% | 0% | 0% | High | Include |
| Adebowale 2020 [98] | 81 | Y | Y | Y | Y | Y | Y | Y | Y | Y | 9 | 100% | 0% | 0% | High | Include |
| Bedekelabou 2022 [99] | 75 | Y | Y | Y | Y | Y | Y | Y | Y | Y | 9 | 100% | 0% | 0% | High | Include |
| Athliamai 2025 [100] | 46 | Y | Y | Y | Y | Y | Y | Y | Y | Y | 9 | 100% | 0% | 0% | High | Include |
| Larbi 2022 [58] | 36 | Y | Y | Y | Y | Y | Y | Y | Y | Y | 9 | 100% | 0% | 0% | High | Include |
| Ojo 2019 [101] | 19 | Y | Y | Y | Y | Y | Y | Y | Y | Y | 9 | 100% | 0% | 0% | High | Include |
| Adebowale 2021 [102] | 56 | Y | Y | Y | Y | Y | Y | Y | Y | Y | 9 | 100% | 0% | 0% | High | Include |
| Adebowale 2022 [103] | 81 | Y | Y | Y | Y | Y | Y | Y | Y | Y | 9 | 100% | 0% | 0% | High | Include |
| Aguidissou 2020 [104] | 91 | Y | Y | Y | Y | Y | Y | Y | Y | Y | 9 | 100% | 0% | 0% | High | Include |
| Awogbemi 2018 | 36 | Y | Y | Y | Y | Y | Y | Y | Y | Y | 9 | 100% | 0% | 0% | High | Include |
| Bamidele 2022 [105] | 91 | Y | Y | Y | Y | Y | Y | Y | Y | Y | 9 | 100% | 0% | 0% | High | Include |
| Bedekelabou 2022 [99] | 100 | Y | Y | Y | Y | Y | Y | Y | Y | Y | 9 | 100% | 0% | 0% | High | Include |
| Ezeh 2023 [47] | 50 | Y | Y | Y | Y | Y | Y | Y | Y | Y | 9 | 100% | 0% | 0% | High | Include |
| Hambolu 2024 [106] | 55 | Y | Y | Y | Y | Y | Y | Y | Y | Y | 9 | 100% | 0% | 0% | High | Include |
| Jibril 2021 [52] | 91 | Y | Y | Y | Y | Y | Y | Y | Y | Y | 9 | 100% | 0% | 0% | High | Include |
| Okwundu 2025 [107] | 86 | Y | Y | Y | Y | Y | Y | Y | Y | Y | 9 | 100% | 0% | 0% | High | Include |
| Oloso 2019 [68] | 18 | Y | Y | Y | Y | Y | Y | Y | Y | Y | 9 | 100% | 0% | 0% | High | Include |
| Ejikeugwu 2025 [108] | 18 | Y | Y | Y | Y | Y | Y | Y | Y | Y | 9 | 100% | 0% | 0% | High | Include |

**Legend:**

Q1. Was the sample frame appropriate to address the target population?

Q2. Were study participants sampled in an appropriate way?

Q3. Was the sample size adequate?

Q4. Were the study subjects and the setting described in detail?

Q5. Was the data analysis conducted with sufficient coverage of the identified sample?

Q6. Were valid methods used for the identification of the condition?

Q7. Was the condition measured in a standard, reliable way for all participants?

Q8. Was there appropriate statistical analysis?

Q9. Was the response rate adequate, and if not, was the low response rate managed appropriately?

**Note:** participants in the context of this study, this refers to livestock animals.

Total Sample Size refers to the total number of individual specimens analysed in each study to determine bacterial prevalence.

Only studies meeting the predefined eligibility criteria for quantitative synthesis were assessed and are presented in this table.

**References**

1. Dougnon P, Dougnon V, Legba B, Fabiyi K, Soha A, Koudokpon H, et al. Antibiotic profiling of multidrug resistant pathogens in one ‑ day ‑ old chicks imported from Belgium to benin. BMC Vet Res. 2023;:1–8. https://doi.org/10.1186/s12917-023-03570-y.

2. Fashae K, Engelmann I, Monecke S, Braun SD, Ehricht R. Molecular characterisation of extended-spectrum ß-lactamase producing Escherichia coli in wild birds and cattle, Ibadan, Nigeria. BMC Vet Res. 2021;17:1–12. https://doi.org/10.1186/s12917-020-02734-4.

3. Beshiru A, Igbinosa IH, Igbinosa EO. An Investigation on Antibiogram Characteristics of Escherichia coli Isolated From Piggery Farms in Benin City , Nigeria. 2016;1:8–12.

4. Abubakar, M.B., Salihu, M.D,, Aliyu, R.M.1, Bello, A., Tukur, H. and Shuaibu AB. 1Department. Occurrence and Antimicrobial resistance of ESBL- producing Escherichia coli in Indigenous Chickens and retailed Table-Eggs in Sokoto Metropolis , Nigeria. 2016;5 December:56–60.

5. ABUBAKAR S. M. ABBA, YA ADAMU, SIRAJO GARBA NAFIU LAWAL AAHJ. MOLECULAR DETECTION OF RESISTANCE IN ESCHERICHIA COLI ISOLATED IN DISEASED CHICKENS FROM COMMERCIAL FARMS IN NORTHWEST, NIGERIA. 2025;68:20–43.

6. Shittu AO, Taiwo FF, Froböse NJ, Schwartbeck B, Niemann S, Mellmann A, et al. Genomic analysis of Staphylococcus aureus from the West African Dwarf ( WAD ) goat in Nigeria. Antimicrob Resist Infect Control. 2021;:1–12. https://doi.org/10.1186/s13756-021-00987-8.

7. Adebowale O, Makanjuola M, Bankole N, Olasoju M, Alamu A, Kperegbeyi E, et al. Multi-Drug Resistant Escherichia coli , Biosecurity and. 2022.

8. Adeluwoye-ajayi OA, Thomas FM, Awoniyi RR. Detection of Extended Spectrum Beta-Lactamase Production in Escherichia coli Isolated from Cattle Faeces in Owo Metropolis. 2021;8:32–9. https://doi.org/10.9734/IJPR/2021/v8i430212.

9. Jackson CR, Oluwadun A, Iwalokun BA, Adenipekun EO, Frye JG, Barrett JB, et al. Prevalence and Antimicrobial Resistance in Escherichia coli from Food Animals in Lagos, Nigeria. 2015;00:1–8. https://doi.org/10.1089/mdr.2014.0222.

10. Agbankpe AJ, Kougblenou SD, Dougnon TV, Oussou A, Gbotche E, Koudokpon CH, et al. Prevalence and Antimicrobial Resistance of Campylobacter coli and Campylobacter jejuni Isolated from Pig Guts , Pig Feces , and Surface Swabs from the Cutting Tables at Slaughterhouse and Taverns in Southern Benin. 2022;2022.

11. Okwundu I, Johnny E. PREVALENCE OF MULTI-DRUG RESISTANT ESCHERICHIA COLI AND THE RISK PRACTICES FOR SPREAD IN POULTRY FARMS AND LIVE BIRD MARKET IN NSUKKA , ENUGU STATE , NIGERIA. 2025;22:5878–88.

12. Al-mustapha AI, Adisa I, Adeolu O. Antibiotic resistance genes , mobile elements , virulence genes , and phages in cultivated ESBL-producing Escherichia coli of poultry origin in Kwara State , North Central Nigeria International Journal of Food Microbiology Antibiotic resistance genes , mo. Int J Food Microbiol. 2023;389 March:110086. https://doi.org/10.1016/j.ijfoodmicro.2023.110086.

13. Abraham A, Stella S, Muinah F, Oreoluwa BI, Julien K. Plasmid pro fi le and role in virulence of salmonella enterica serovars isolated from food animals and humans in Lagos Nigeria. Pathog Glob Health. 2019;113:282–7. https://doi.org/10.1080/20477724.2019.1691364.

14. Adedokun FL, Ajayi A, Essiet UU, Oduyebo O, Adeleye AI, Smith SI. Antibiotic Resistance and Plasmid Replicon Types of Non-Typhoidal Salmonella Serovars Isolated From Food Animals and Humans in Lagos , Nigeria. 2023;:4–9. https://doi.org/10.1177/11786361231181909.

15. Ajibola AT, Lagarde M De, Ojo OE, Balogun SA, Vanier G, Fairbrother JM, et al. Antimicrobial resistance and virulence gene profiles of Escherichia coli isolated from poultry farms using One Health perspective in Abeokuta , Nigeria. 2025.

16. Akanbi OB, Olorunshola ID, Osilojo P, Ademola E. Escherichia coli Infections , and Antimicrobial Resistance In Poultry Flocks , in North Central Nigeria. 2022;:8–9. https://doi.org/10.20473/mkh.v33i3.2022.188-207.

17. Ahmed AO, Raji MA, Mamman PH, Kwanashie CN, Raufu A, Akorede GJ, et al. Salmonella enterica isolated from selected poultry farms in Kwara State , Nigeria between 2015 and 2016 showed resistance to critical antimicrobials. 2020;21:14–24.

18. Ahmed AO, Ameh JA, Abubakar MB, Raufu IA. Campylobacteriosis in Africa : A neglected demon. The Microbe. 2025;7 May:100409. https://doi.org/10.1016/j.microb.2025.100409.

19. Sosthène D, Akre T, Okou OC, Koffi AE, Benjamin K, Auguste J, et al. Prévalence et phénotypes de résistances aux antibiotiques des souches d ’ Escherichia coli responsables des diarrhées de veaux à Daloa , Côte d ’ Ivoire Prevalence and antibiotic resistance patterns of Escherichia coli strains causing calves diarrhea in D. 2022;16 December:2685–98.

20. Amaechi, N and Nwankwo IU. Evaluation of Prevalence and Antimicrobial Resistance using Enterococci Isolates from Pigs and Poultry Birds in Abia State , Nigeria. 2015;4:825–33.

21. Anyanwu MU, Ugwu IC, Okorie- OJ, Aneke I, Chah KF. Sorbitol non- ­ fermenting Escherichia coli and E . coli O157 : prevalence and antimicrobial resistance profile of strains in slaughtered food animals in Southeast Nigeria. 2022;:1–8. https://doi.org/10.1099/acmi.0.000433.

22. T. O. ARIOM, K. O. BELLO, 3I. A. KEHINDE 2A. O., ATAYESE, 2G. O. OLANREWAJU A 2N. DA. Klebsiella pneumoniae IN SMALLHOLDER POULTRY. 2025;:41–53.

23. Assoumy MA, Bedekelabou AP, Teko-agbo A, Ossebi W, Akoda K. Antibiotic resistance of Escherichia coli and Salmonella spp . strains isolated from healthy poultry farms in the districts of Abidjan and Agnibilékrou ( Côte d ’ Ivoire ). 2021;14.

24. Aworh MK, Kwaga J, Okolocha E, Harden L, Hull D, Hendriksen RS, et al. Extended-spectrum ß-lactamase-producing Escherichia coli among humans, chickens and poultry environments in Abuja, Nigeria. One Heal Outlook. 2020;2. https://doi.org/10.1186/s42522-020-00014-7.

25. Aworh MK, Ekeng E, Nilsson P, Egyir B, Owusu-Nyantakyi C, Hendriksen RS. Extended-Spectrum ß-Lactamase-Producing Escherichia coli Among Humans, Beef Cattle, and Abattoir Environments in Nigeria. Front Cell Infect Microbiol. 2022;12 April:1–11. https://doi.org/10.3389/fcimb.2022.869314.

26. Ayeni FA, Olujobi OF. A Preliminary Investigation of Prevalence of Extended Spectrum Beta Lactamases among Enterobacteriaceae Isolated from Poultry Farms in. 2015;:46–51.

27. Bamidele O, Yakubu A, Joseph EB, Amole TA. Antibiotic Resistance of Bacterial Isolates from Smallholder Poultry Droppings in the Guinea Savanna Zone of Nigeria. 2022;:1–18.

28. Bedekelabou AP, Talaki E, Dolou M, Diouf A, Alambedji RB. Antibiotic resistance of enterobacteria ( Escherichia coli , Klebsiella spp . and Salmonella spp ) isolated from healthy poultry and pig farms in peri-urban area of Lome , Togo. 2020;14:657–66. https://doi.org/10.5897/AJMR2020.9437.

29. Beshiru A, Igbinosa IH, Akinnibosun O, Ogofure AG, Okafor AD, Uwhuba KE, et al. Characterization of resistance and virulence factors in livestock ‑ associated methicillin ‑ resistant Staphylococcus aureus. Sci Rep. 2024;:1–11. https://doi.org/10.1038/s41598-024-63963-3.

30. Bonkoungou I, Ouindgueta, Edith M, Nikiema M, Garba Z, Bako E, et al. Detection of bla CTX-M , bla TEM , and bla SHV genes in ESBL- producing enterobacterales from poultry farms in the peri-urban area of Ouagadougou , Burkina Faso. 2025;17 June:14–22. https://doi.org/10.5897/JMA2024.0472.

31. Bouda SC, Kagambèga A, Bonifait L, Gall F Le, Ibrahim HB, Bako E, et al. Prevalence and Antimicrobial Resistance of Salmonella enterica Isolated from Chicken and Guinea Fowl in Burkina Faso. 2019;4:64–71. https://doi.org/10.11648/j.ijmb.20190403.12.

32. Ifeanyichukwu I, O AC, Malachy U, Peter E, S IC, Lilian O. Multiple Antibiotic Resistance , Antibiogram and Phenotypic Detection of Metallo-Beta- Lactamase ( MBL ) from Escherichia coli of Poultry Origin Abstract. 2017;:1–5. https://doi.org/10.21767/2576-1412.100015.

33. Ejikeugwu C, Esimone C, Iroha I, Eze P, Adikwu M. Genotypic and Phenotypic Characterization of MBL Genes in Pseudomonas aeruginosa Isolates from the Non-hospital Environment. 2018;12:1877–85.

34. Julien CK, René YK, Komissiri D, Bertin TK, Eugène KK, Abraham A, et al. Phenotype and Molecular Characterization of Antibiotic Resistance of Salmonella spp . from Cattle in Abidjan District , Côte d ’ Ivoire. 2019;18 September 2016:1–10. https://doi.org/10.9734/JAMB/2019/v18i430176.

35. Isaiah DO. Multiple antibiotic resistance indexing and molecular identification of Escherichia coli isolated from clinical and nonclinical sources in Port Harcourt Metropolis , Nigeria. 2025;:1–12.

36. Egbule OS, Yusuf I. Multiple Antibiotic Resistances in Escherichia coli Isolated from Cattle and Poultry Faeces in Abraka , South-South Nigeria. 2019;42:585–94.

37. Ejeh FE, Lawan FA, Abdulsalam H, Mamman PH, Kwanashie CN. Multiple antimicrobial resistance of Escherichia coli and Salmonella species isolated from broilers and local chickens retailed along the roadside in Zaria , Nigeria. 2017;15:45–53.

38. Chika E, Ifeanyichukwu I, Carissa D, Thomas A, Okoro O, Joshua E, et al. Occurrence of Metallo-Beta-Lactamase-Producing Enterobacteriaceae from a Local Poultry Farm in Abakaliki, Nigeria. 2016;1:70–5.

39. Ejikeugwu C, Nworie O, Saki M, Al-dahmoshi HOM, Al-khafaji NSK, Ezeador C, et al. Metallo- β -lactamase and AmpC genes in Escherichia coli , Klebsiella pneumoniae , and Pseudomonas aeruginosa isolates from abattoir and poultry origin in Nigeria. 2021;:1–9.

40. Agoba EE, Adu F, Agyare C, Boamah VE. Antibiotic resistance patterns of bacterial isolates from hatcheries and selected fish farms in the Ashanti region of Ghana. 2017;9 August:35–46. https://doi.org/10.5897/JMA2017.0387.

41. Oluwabunmi I, Id F, Ramon E, Lettini AA, Muhammad M, Longo A, et al. Assessing the mechanisms of multi-drug resistant non-typhoidal Salmonella ( NTS ) serovars isolated from layer chicken farms in Nigeria. 2023;:1–19. https://doi.org/10.1371/journal.pone.0290754.

42. Raufu IA, Ahmed OA, Aremu A, Ameh JA, Timme RE, Hendriksen RS, et al. Occurrence , antimicrobial resistance and whole genome sequence analysis of Salmonella serovars from pig farms in Ilorin , North-central Nigeria. Int J Food Microbiol. 2021;350 December 2020:109245. https://doi.org/10.1016/j.ijfoodmicro.2021.109245.

43. Gaddafi MS, Yakubu Y, Bello MB, Lawal H, Bitrus AA, Musawa IA, et al. Prevalence of Methicillin-resistant Staphylococcus aureus in Livestock Production System of Nigeria : A Systematic Review. 2024. https://doi.org/10.5152/actavet.2024.24009.

44. Gaddafi MS., , Yakubu Y., , Bello, M. B.3 , Bitrus, A. A.4 , Musawa AI., Garba B., , Lawal H., , Aliyu, M. A.1 , Barka, S. A.5, Emeka AJ. OCCURRENCE AND ANTIBIOTIC RESISTANCE PROFILES OF METHICILLIN-RESISTANT STAPHYLOCOCCUS AUREUS ( MRSA ) IN LAYER CHICKENS IN KEBBI , NIGERIA. 2022;:37–45. https://doi.org/10.2478/fv-2022-0016.

45. Gbanamou W, Omotosho OO, Anifowose OR, Olukole SG. Characterization and Antibiogram of Escherichia coli Isolated from Indigenous Chickens in Ibadan , Nigeria. 2024;:240–8. https://doi.org/10.17957/IJAB/15.2198.

46. Gbonon M, Carole V, Kouadio GN. Antimicrobial Resistance Profile and Molecular Characterization of Extended- spectrum Beta-lactamase Genes in Enterobacteria Isolated from Human , Antimicrobial Resistance Profile and Molecular Characterization of Extended-spectrum Beta- lactamase Genes i. 2018; May. https://doi.org/10.9734/JAMB/2018/39955.

47. Ezeh GC, Ogugua AJ, Anyanwu MU, Awoyomi OJ, Nwanta JA. Prevalence , Antimicrobial Resistance , and Virulence Determinants of Enterococci in Poultries in Nsukka , Enugu State , Nigeria. 2023;49:26–35. https://doi.org/10.5152/actavet.2022.22069.

48. Ibrahim T, Ngwai YB, Pennap GRI, Ishaleku D, Tsaku PA. Antimicrobial Resistance Profile of Salmonella Typhimurium Isolated from Commercial Poultry and Poultry Farm Handlers in Nasarawa State , Nigeria. 2019;28 November 2017:1–12. https://doi.org/10.9734/MRJI/2019/v28i430136.

49. Farms P, State E, Beshiru A, Ikediashi SC, Igbinosa EO. Identification and Characterization of Salmonella Serovars Isolated from Pig Farms in Benin City, Edo State, Nigeria: One Health Perspective 1,2. 2020;00:1–10. https://doi.org/10.1089/mdr.2019.0357.

50. Kwoji ID, Jauro S, Musa JA, Lekko YM, Salihu SI. Phenotypic detection of methicillin-resistant Staphylococcus aureus in village chickens from poultry markets in Maiduguri , Nigeria. 2019;7710 June:163–7.

51. Isichei-Ukah, B. O., *1, 3Akinnibosun, O., 1Nwaka, C. N., and 1, 2Igbinosa EO. A survey of antibiotic resistance and virulence factors in Enterococcus species isolated from poultry farms in Benin City , Nigeria Abstract : Une enquête sur la résistance aux antibiotiques et les facteurs de virulence chez l. 2024;25:75–85.

52. Jibril AH, Okeke IN, Dalsgaard A, Garc V, Olsen JE. Genomic Analysis of Antimicrobial Resistance and Resistance Plasmids in Salmonella Serovars from Poultry in Nigeria. 2021;:1–21.

53. Karikari AB, Obiri-danso K, Frimpong EH, Krogfelt KA. Antibiotic Resistance of Campylobacter Recovered from Faeces and Carcasses of Healthy Livestock. 2017;2017.

54. Amosun BE, Ola-daniel F. Escherichia coli O157 : H7 and Salmonella species isolated from Ducks and Indigenous chickens. 2022; February. https://doi.org/10.12834/VetIt.2553.16733.2.

55. Nadège K, Mireille KS, René KA, Aubin T, Antoinette AA, Thomas DA. Shiga toxigenic , enteroinvasive and enteropathogenic Escherichia coli in fish from experimental fish farm ( Layo ), Côte d ’ Ivoire. 2019;13:369–75. https://doi.org/10.5897/AJMR2019.9105.

56. Kyrian-ogbonna E, Ekwealor IA, Eluba A, Nworji E. Antibiotic Resistance Patterns of Escherichia coli Detected in Pig Faecal Samples from Anambra State Antibiotic Resistance Patterns of Escherichia coli Detected in Pig Faecal Samples from Anambra State. 2021; October. https://doi.org/10.9734/MRJI/2021/v31i530320.

57. Larbi RO, Ofori LA, Sylverken AA, Ayim-Akonor M, Obiri-Danso K, Ohene Larbi R, et al. Antimicrobial Resistance of Escherichia coli from Broilers, Pigs, and Cattle in the Greater Kumasi Metropolis, Ghana. Int J Microbiol. 2021;2021. https://doi.org/10.1155/2021/5158185.

58. Larbi RO, Adeapena W, Ayim-akonor M, Ansa EDO, Tweya H, Terry RF, et al. Antimicrobial , Multi-Drug and Colistin Resistance in Enterobacteriaceae in Healthy Pigs in the Greater Accra Region of Ghana , 2022 : A Cross-Sectional Study. 2022.

59. Mansaray AHD, Yankson DPY, Johnson RAB, Moses FL, Kanu JS, Kamara IF, et al. Bacterial Isolates and Antibiotic Resistance of Escherichia coli Isolated from Fresh Poultry Excreta Used for Vegetable Farming in Freetown , Sierra Leone. 2022; ii.

60. Ayim-akonor M, Larbi RO, Owusu-ntumy D Dela, Sasu BK, Emefa H, Asa O, et al. Antimicrobial Susceptibility Profile and Extended-Spectrum Beta-Lactamase Phenotype of E . coli Isolated From Poultry. 2025;2025.

61. Sati NM, Card RM, Barco L, Muhammad M, Luka PD, Chisnall T, et al. Antimicrobial Resistance and Phylogenetic Relatedness of Salmonella Serovars in Indigenous Poultry and Their Drinking Water Sources in North Central Nigeria. 2024.

62. Ngbede EO, Raji MA, Kwanashie CN, Kwada J, Kwaga P. Antimicrobial resistance and virulence profile of enterococci isolated from poultry and cattle sources in Nigeria. Trop Anim Health Prod. 2016. https://doi.org/10.1007/s11250-016-1212-5.

63. Njoga EO, Ochi E, Nwobi OC, Ugwunwarua JC, Anidobe EC. Fecal carriage and multidrug resistance profiles of zoonotic Campylobacter species isolated from broilers in Nsukka , Nigeria. 2025;18:3409–19. https://doi.org/10.14202/vetworld.2025.3409-3419.

64. Okorafor ON, Anyanwu MU, Nwafor EO, Anosa GN. Multidrug-resistant enterobacteria colonize commercial day-old broiler chicks in Nigeria. 2019;12:418–23. https://doi.org/10.14202/vetworld.2019.418-423.

65. Ogefere HO, Agbe SO, Ibadin EE. Detection of Extended Spectrum Beta-Lactamases Among Gram Negative Bacilli Recovered from Cattle Feces in Benin City , Nigeria. 2017;9 May:177–81. https://doi.org/10.15835/nsb9210005.

66. Okoye SC, Chukwunwejim CR, Ekpunobi NF, Ajasa OS, Israel O, Obidi N, et al. Prevalence of Multidrug-Resistant Escherichia coli O157 : H7 in Poultry Faeces from Live Bird Markets in Lagos , Nigeria. 2025.

67. Gabriel O, Olufisayo O, Remilekun O, Chris O, Taiwo O. ANTIBIOTIC RESISTANCE IN CRITICALLY ILL CATTLE AT A MAJOR MARKET AND ABATTOIR IN IBADAN , NIGERIA. 2025;22:6217–27.

68. Oloso NO, Adeyemo IA, Heerden H Van. Antimicrobial Drug Administration and Antimicrobial Resistance of Salmonella Isolates Originating from the Broiler Production Value Chain. 2019.

69. Olu-taiwo MA, Egyir B, Owusu-nyantakyi C, Forson AO, Opintan JA. Molecular characterization of multidrug- resistant Escherichia coli in the Greater Accra Region , Ghana : a ‘ One Health ’ approach. 2025.

70. Shittu OB, Uzairue LI, Ojo OE, Obuotor TM, Folorunso JB, Raheem- RR, et al. Antimicrobial resistance and virulence genes in Salmonella enterica serovars isolated from droppings of layer chicken in two farms in Nigeria. 2022; February:3891–906. https://doi.org/10.1111/jam.15477.

71. Adetunji VO, Davies A, Chisnall T, Ndahi MD, Fagbamila IO, Ekeng E, et al. Genomic Diversity and Antibiotic Resistance of Escherichia coli and Salmonella from Poultry Farms in Oyo State , Nigeria. 2025;:1–12.

72. Omoshaba EO, Ojo OE, Oyekunle MA, Sonibare AO, Adebayo AO. Methicillin-resistant Staphylococcus aureus ( MRSA ) isolated from raw milk and nasal swabs of small ruminants in Abeokuta , Nigeria. 2020.

73. Peter E, Chinaemerem ANI, John O, Victor O, Sunday O, Glory O, et al. Prevalence and Risk Factors of Extended- spectrum β -Lactamase- Producing Escherichia coli from Poultry Flocks in Benin City , Nigeria. 2024;18:66–74.

74. Paintsil EK, Ofori LA, Akenten CW, Zautner AE, Mbwana J, Khan NA, et al. Antibiotic ‑ Resistant Arcobacter spp . in commercial and smallholder farm animals in Asante Akim North Municipality , Ghana and Korogwe Town Council , Tanzania : a cross ‑ sectional study. Gut Pathog. 2023;:1–11. https://doi.org/10.1186/s13099-023-00588-3.

75. Raji MA, Kazeem HM, Magyigbe KA, Ahmed AO, Lawal DN, Raufu IA. Salmonella Serovars , Antibiotic Resistance , and Virulence Factors Isolated from Intestinal Content of Slaughtered Chickens and Ready-to-Eat Chicken Gizzards in the Ilorin Metropolis , Kwara State , Nigeria. 2021;2021. https://doi.org/10.1155/2021/8872137.

76. Sanou S, Ouedraogo AS, Lounnas M, Zougmore A, Pooda A, Zoungrana J, et al. Epidemiology and molecular characterization of Enterobacteriaceae producing extended-spectrum β-lactamase in intensive and extensive breeding animals in Burkina Faso . PAMJ - One Heal. 2022;8:1–12. https://doi.org/10.11604/pamj-oh.2022.8.4.33553.

77. Sekyere JO, Adu F. Prevalence of Multidrug Resistance among Salmonella enterica Serovar Typhimurium Isolated from Pig Faeces in Ashanti Region , Ghana. 2015;2015. https://doi.org/10.1155/2015/898790.

78. Phenotypique C, Resistance DELA, Chez I, Humains LES, Animaux LES, Dans ET. Monsieur Mamadou SIDIBE. 2020.

79. Soma D, Diarra FBJ, Bonkoungou IJO, Somda NS. Assessing antibiotic use practices on central Burkina Faso cattle farms and the associated risks to environmental and human health contamination: A pilot study. 2025;11:1–12. https://doi.org/10.14202/IJOH.2025.1-12.

80. Soré S, Bintou F, Diarra J, Sampo E. Knowledge , perception and beliefs of human health workers and veterinarians on antimicrobial resistance in Ouagadougou , Burkina Faso. 2022;16:19–26. https://doi.org/10.5897/AJPP2021.5290.

81. Tahou EJ, Kouadio KI, Ouattara MB, Touvoli AM, Guetta AS-PN, Guessennd KN. Prevalence and antibiotic resistance in Escherichia coli strains isolated from poultry farms in Azaguie , Côte d ’ Ivoire. 2025.

82. Tama SC, Ngwai Y, Pennap G, Nkene I. Antimicrobial Resistance Profile and Extended Spectrum Beta-Lactamase Resistance Genes in Escherichia coli from Poultry Droppings in Nasarawa , Antimicrobial Resistance Profile and Extended Spectrum Beta-Lactamase Resistance Genes in Escherichia coli from. 2021; July. https://doi.org/10.9734/ajbgmb/2021/v8i230192.

83. Ishola OO. Occurrence and antimicrobial susceptibility patterns of Salmonella species from poultry farms in Ibadan , Nigeria. 2022;:1–6.

84. Ugbo EN, Effendi MH, Witaningrum AM, Tyasningsih W, Agumah BN, Ugbo AI, et al. Antimicrobial resistance pattern of Salmonella spp . isolated from poultry farms in Abakaliki , Nigeria. 2023;24:5207–14. https://doi.org/10.13057/biodiv/d240965.

85. Ugbo EN, Effendi MH, Ugbo AI, Tyasningsih W, Agumah BN, Raharjo HM, et al. Molecular identification of tetracycline resistance genes in Escherichia coli isolates from internal organs of swine sold on Abakaliki , Nigeria. 2025;15:171–8. https://doi.org/10.5455/OVJ.2025.v15.i1.16.

86. Diarrhoea C, Mc U, Gi E, Cp E, Okezie U, So E. Clinical Microbiology : Open Access Antibiotic Susceptibility Profile of Escherichia coli and Salmonella Causing. 2017;6:2–6. https://doi.org/10.4172/2327-5073.1000277.

87. Chukwudile UB, Ugwu CC. Molecular Determinants of Virulence and Antimicrobial Resistance among Enterococcus Species Isolated from Chickens. 2023;6604:47–57. https://doi.org/10.22069/psj.2022.20176.1817.

88. Vounba P, Kane Y, Ndiaye C, Arsenault J. Molecular Characterization of Escherichia coli Isolated from Chickens with Colibacillosis in Senegal. 2018;15:517–25. https://doi.org/10.1089/fpd.2017.2394.

89. Vounba P, Arsenault J, Bada-alambe R, Fairbrother JM. Antimicrobial Resistance and Potential Pathogenicity of Escherichia coli Isolates from Healthy Broilers in Que ´bec, Canada. Microb Drug Resist. 2019;25:1111–21. https://doi.org/10.1089/mdr.2018.0403.

90. Yao RK, Coulibaly JK, Tiekoura BK, Yapi FH, Djaman JA. Molecular Characterisation of Extended-Spectrum Beta-lactamase Producing Escherichia coli Isolated from Cattle Faeces in Abidjan District, Ivory Coast. Microbiol Res J Int. 2018;25:1–10. https://doi.org/10.9734/MRJI/2018/45271.

91. Yhiler NY, Bassey BE, Paul I, Francis UM, Anne A, Okocha-ejeko A. Antimicrobial resistance pattern in Salmonella enterica from clinical and poultry sources in Calabar , Nigeria. 2019;11 December:5–10. https://doi.org/10.5897/JMA2019.0413.

92. Abubakar Y, Yakubu Y, Garba B, Lawal N, Dahiru UM. Occurrence and Antimicrobial Resistance Profile of Escherichia coli in Poultry from Gusau metropolis , Nigeria. 2023;5:28–38. https://doi.org/10.36108/jvbs/3202.50.0240.

93. Bodombossou EM, Agbessi K, Comlan R, Tchaou I, Akapko C, Armand L, et al. Formal and informal antimicrobial trade and usage in farmed animals of the urban area of Lom ´ e , Togo. 2025;21 September. https://doi.org/10.1016/j.onehlt.2025.101195.

94. Cama E, Krumkamp R, Mawuli L, Zakane A, Jahn A, Sié A, et al. Exploring households ’ access and utilization of antibiotics for their livestock : A cross-sectional study in Ghana and Burkina Faso. J Glob Antimicrob Resist. 2025;44:30–9. https://doi.org/10.1016/j.jgar.2025.04.020.

95. Sawadogo A, Kagambèga A, Moodley A, Ouedraogo AA, Barro N, Dione M. Knowledge, Attitudes, and Practices Related to Antibiotic Use and Antibiotic Resistance among Poultry Farmers in Urban and Peri-Urban Areas of Ouagadougou, Burkina Faso. Antibiotics. 2023;12. https://doi.org/10.3390/antibiotics12010133.

96. Alhaji N, Adamu A, Odetokun I, Lawan M, Fasina FO. Practices of antimicrobial usage and associated resistance emergence in smallholder beef cattle production systems in Northern Nigeria : Drivers and One Health challenge. 2022;:1–12.

97. Adelowo OO, Caucci S, Banjo OA, Nnanna OC, Awotipe EO, Peters FB, et al. Extended Spectrum Beta-Lactamase ( ESBL ) -producing bacteria isolated from hospital wastewaters , rivers and aquaculture sources in Nigeria. 2017.

98. Adebowale OO, Adeyemo FA, Bankole N. Farmers ’ Perceptions and Drivers of Antimicrobial Use and Abuse in Commercial Pig Production ,. Int J Environ Res Public Heal. 2020;17. https://doi.org/doi:10.3390/ijerph17103579.

99. Bedekelabou AP, Talaki E, Dzogbema KFX, Dolou M, Savadogo M, Seko MO, et al. Assessing farm biosecurity and farmers’ knowledge and practices concerning antibiotics and antibiotic resistance in poultry and pig farms in Southern Togo. Vet World. 2022;15:1727–37. https://doi.org/10.14202/vetworld.2022.1727-1737.

100. Bitrus AA, Charibu DH, Akinsola OM. Awareness of antibiotic use and antibiotic resistance among. 2025;23 July 2024:1–17. https://doi.org/10.12982/VIS.2025.068.

101. Balogun SA, Ojo OE, Bayode OO, Kareem SO. High level detection of extended spectrum beta-lactamase gene encoding Enterobacteriaceae in public toilets in Abeokuta , Nigeria. 2020;49:165–72.

102. Adebowale O, Makanjuola M, Bankole N, Olanike A, Awoseyi A, Awoyomi OJ. BIOSECURITY AND ANTIMICROBIAL USE PRACTICES IN LIVE BIRD MARKETS WITHIN ABEOKUTA METROPOLIS , SOUTHWEST , NIGERIA : A PRELIMINARY SURVEY. 2021;44:187–202.

103. Adebowale OO. Porcine circovirus type 2 and its associated diseases in southwestern Nigeria : Farmers ’ perception and level of awareness. 2022;7710 June:203–10.

104. Aguidissou ONC, Boko CK, Adoligbe CM, Dete CH, Capo-chichi PT. Inventory of biosecurity measures and antibiotics therapy practices on laying hen farms in Benin. 2020;13.

105. Bamidele O, Amole TA, Oyewale OA, Bamidele OO, Yakubu A, Ogundu UE, et al. Antimicrobial Usage in Smallholder Poultry Production in Nigeria. 2022;2022.

106. Hambolu DA, Olatoye OI, Besong MA, Call DR. Low ‑ cost biosecurity measures are associated with reduced detection of non ‑ Typhoidal Salmonella in Nigerian poultry while inappropriate antibiotic use is widespread. Sci Rep. 2024;:1–8. https://doi.org/10.1038/s41598-024-72317-y.

107. Okafor UC, Okorie-kanu OJ, Ogugua AJ, Ikeogu CF, Okafor SC, Anyanwu MU, et al. Molecular Epidemiology , Antimicrobial Resistance , and Virulence Profiles of Staphylococcus aureus from Fish , Aquatic Environments , and Fish Handlers in Southeast Nigeria. 2025;:1–25.

108. Ejikeugwu CP, Edeh C, Nwakaeze EA, Adikwu MU, Torres C, Creevey CJ, et al. Whole-Genome Sequencing Uncovers Chromosomal and Plasmid-Borne Multidrug Resistance and Virulence Genes in Poultry-Associated Escherichia coli from Nigeria. 2025;:1–17.
